# How Polymer Chains Remember Their Crystalline Past: Simulations Uncover the Molecular Origin of Melt Memory

**DOI:** 10.1021/jacs.6c13790

**Published:** 2026-08-14

**Authors:** Antonio De Nicola, Alejandro J. Müller, Dario Cavallo, Giuseppe Milano

**Affiliations:** † Department of Chemical, Materials and Production Engineering, 9307 University of Naples Federico II, Piazzale V. Tecchio, 80, Napoli 80125, Italy; ‡ Research Center for Organic Electronics (ROEL), Yamagata University, Jonan 4-3-16, Yonezawa 992-8510, Japan; § POLYMAT and Department of Polymers and Advanced Materials: Physics, Chemistry and Technology, Faculty of Chemistry, University of the Basque Country UPV/EHU, Paseo Manuel de Lardizabal 3, Donostia-San Sebastián 20018, Spain; ∥ IKERBASQUE, Basque Foundation for Science, Plaza Euskadi 5, Bilbao 48009, Spain; ⊥ Department of Chemistry and Industrial Chemistry, 9302 University of Genoa, Via Dodecaneso 31, Genoa 16146, Italy

## Abstract

Melt memory in semicrystalline polymers is the remarkable ability of polymer chains to retain structural information from a prior crystalline state after being heated above the melting temperature. This phenomenon can induce extraordinary self-nucleation and strongly influence crystallization kinetics and final material properties, yet its molecular origin remains unresolved. Here, using molecular dynamics simulations of linear polymer chains in which the strength of nonbonded interchain interactions is systematically tuned, we show that enhanced interchain attractions stabilize nanoscale regions of increased density and extended trans-planar conformations that persist in the melt, as revealed by analyzing the dynamics through a density-field approach. These residual ordered regions in the melt act as self-nuclei upon cooling, providing a molecular explanation for experimental observations of persistent melt memory in polar polymers. By varying a single chemically meaningful parameter, i.e., the strength of interchain attraction, our model bridges weakly interacting polyolefins and polymers with stronger dipolar or hydrogen-bonding interactions, establishing a direct link between molecular cohesion and memory retention. The results demonstrate that melt memory originates from the interaction-mediated survival of localized structural order in the melt rather than from a completely randomized chain state. These findings provide a molecular framework connecting the chemical structure, intermolecular forces, and macroscopic crystallization behavior, offering new principles for controlling polymer solidification and designing semicrystalline materials with tailored properties.

## Graphical abstract

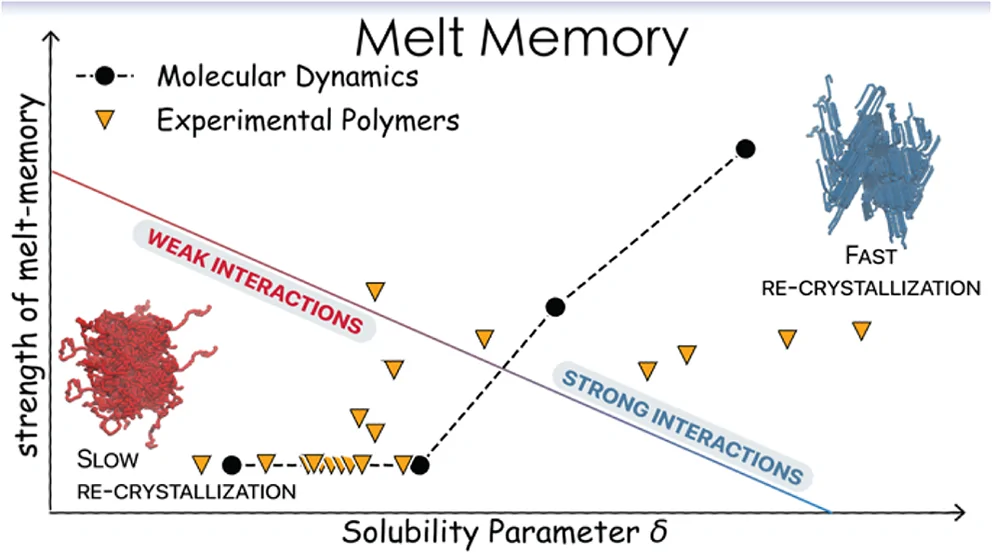

## Introduction

Polymer crystallization is a fascinating subject with many currently open scientific questions. Crystallization controls the mechanical, thermal, and barrier properties of semicrystalline polymers, as well as their biodegradation rates.^1–6^ When a semicrystalline polymer is melted, it can retain the memory of the aligned chains within the original crystals, depending on the temperature and duration of the heating process. This ill-defined residual order, which survives the melting process, can accelerate crystallization upon subsequent cooling via a self-nucleation mechanism.^7^ This behavior is known as “melt memory”, a unique feature of polymeric materials that is not present in low-molecular-mass molecules. Melt memory accelerates the nucleation rate and significantly increases nucleation density, promoting crystallization at much higher temperatures than usual, as well as a refined morphology.^8,9^ In practical terms, this could mean faster processing with much lower energy consumption (as the material solidifies faster). The refined morphology (i.e., significantly smaller spherulitic size that can easily reach diameters lower than 0.4 μm)^7,10,11^ typically leads to an improvement in mechanical properties, enabling the production of more transparent materials with higher impact strength.^12,13^ Although melt memory in polymers has been known for almost 60 years,^14^ its exact origin remains under debate. Notably, melt memory is strongly dependent on chemical structure: nonpolar polyolefins typically show weak or no memory, whereas polymers bearing polar or hydrogen-bonding groups, such as polyamides, exhibit pronounced and persistent melt memory, suggesting that specific intermolecular interactions are the underlying molecular variable.

Self-seeding, on the other hand, occurs when crystals are partially melted, leaving crystal fragments that self-nucleate the polymer (usually through epitaxial nucleation) upon subsequent cooling. After the seminal studies on the self-seeding of polyethylene single crystals by Blundell and Keller,^14,15^ a convenient protocol for investigating self-nucleation via differential scanning calorimetry (DSC) was developed by Lotz et al.,^8^ enabling the study of self-seeding and, more generally, self-nucleation in many bulk polymers.^9,16–21^ The term self-nucleation (SN) is a general term. It encompasses both self-seeding (when the temperature is low enough to leave crystal fragments unmelted) and melt memory (if the temperature is high enough to completely melt the polymer without erasing the memory of the crystalline state).

According to the DSC-based self-nucleation method, a sample in a “standard” semicrystalline state is heated to temperatures that span the melting endotherm and above. After an isothermal holding time at the chosen temperature, denoted as the self-nucleation temperature (*T*_*s*_), the sample is cooled, and its crystallization and subsequent melting are analyzed. When *T*_*s*_ is sufficiently high, melt memory is erased, and no changes are observed in the crystallization temperature upon cooling. The melt is *isotropic,* and the corresponding temperature range is defined as the ***Melting Domain or Domain I***. When *T*_*s*_ is lowered, a temperature region is entered in which subsequent crystallization occurs at higher temperatures, i.e., the ***Self-Nucleation Domain or Domain II***. Müller et al.^7,10^ further divide *Domain II* into two sub-*Domains* according to the *T*_*s*_ temperatures employed. ***Domain IIa, or the Melt Memory Domain***, is defined as the temperature range in which *T*_*s*_ is high enough to cause complete melting of the crystals without transforming the material into an isotropic melt. ***Domain IIb, or the Self-Seeding Domain,*** is defined as the temperature range where *T*_*s*_ is low enough to leave crystal fragments present in the melt, which can act as self-seeds. Finally, if the self-nucleation temperature is even lower, only partial melting occurs, and the unmolten crystals can be annealed during the time spent at *T*_*s*_. This lower temperature range is defined as the ***Self-Nucleation and Annealing Domain, also known as Domain III.*** Figure 1 illustrates the different SN *Domains* and their locations relative to the standard melting endotherm of a given polymer, along with cartoons representing idealized molecular descriptions. On the right-hand side of the *y*-axis, the crystallization temperatures are plotted as a function of *T*_*s*_ (*x*-axis).

**Figure 1:**
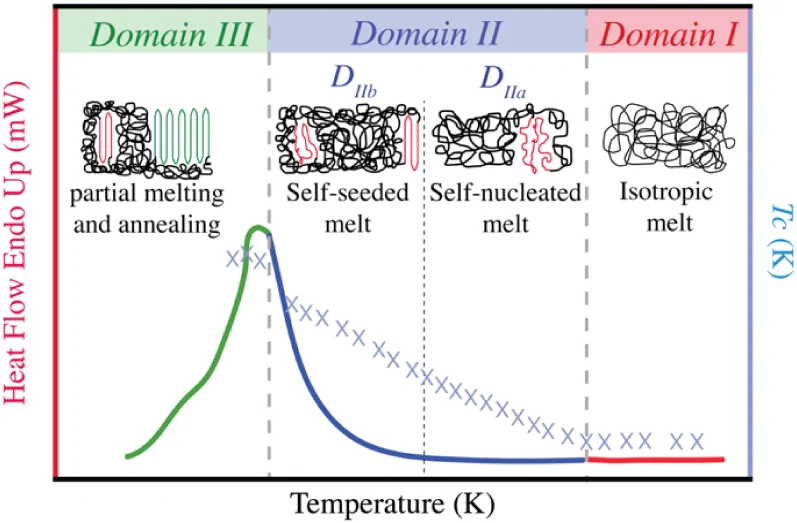
Schematic representation of SN *Domains* on a representative DSC melting scan. Light-blue cross data refer to the crystallization temperature, *T_c_*, on the right *y*-axis. More details can be obtained in refs 6,9 .

Regarding the origin of melt memory, that is, self-nucleation in *Domain IIa*, two specific classes of polymers must be distinguished: random copolymers and homopolymers. In random copolymers, the crystallization process leads to segregation of the counits, which are fully or partially excluded from the crystalline lattice, into the amorphous phase. This generates a complex chain topology in the amorphous regions, hindering isotropization of the crystalline stems upon melting and, consequently, enhancing the recrystallization kinetics upon cooling.^22–27^ The melt memory temperature region in random copolymers can also extend above the equilibrium melting temperature.^22^

In the case of homopolymers, two prerequisites are required to observe melt memory: a molecular weight exceeding a critical value^30,31^ and sufficiently strong intermolecular interactions among the chains.^28,29^ Linear polyethylene exhibits no melt memory, not even at ultrahigh molecular weights.^30^ Instead, “polar” homopolymers, such as polyethers, polyesters, polycarbonates, polyamides, poly­(ester–amides),^28,29,31,32^ and fluoropolymers^33^ show a persistence of melt memory at temperatures that increase with the extent of intermolecular interactions, above a certain threshold (quantified, for instance, by means of the solubility parameter of the repeating unit^28^ or by the dielectric constant of the polymer^33^).

Given that obtaining direct experimental evidence about the nature of the residual order that provokes melt memory is challenging, if not impossible,^18,22^ Molecular Dynamics (MD) simulations stand out as a valuable tool for gaining insights into the phenomenon. Despite the extensive body of experimental literature on melt memory, few works have addressed the problem from a simulation perspective.^34,35^ In particular, melt memory in random copolymers has been successfully captured via dynamic Monte Carlo simulations and attributed to the demixing between crystallizable and noncrystallizable sequences during the first crystallization process.^34^ Moreover, melt memory was related to the slow changes in the “local” entanglement length, as determined using MD simulations and primitive path analysis.^35^ Only recently, Yamamoto et al. investigated the mechanical properties of recrystallized polyethylene that had undergone different recrystallization processes.^36^ On the other hand, the effect of intermolecular interaction strength in polar homopolymers on melt memory remains unexplored in simulations.

This study, for the first time, investigates the melt memory of homopolymers with varying degrees of chain interaction using MD simulation. It is shown that a small fraction of chain segments with “local” density higher than the average and close to that of a perfect crystal can persist above the polymer’s macroscopic melting temperature. The persistence temperature increases with stronger intermolecular interactions. Recrystallization upon cooling is clearly accelerated and initiated by these residual-ordered regions, especially for chains where intermolecular interactions exceed a certain threshold, thereby successfully reproducing the experimental results reported in the literature.

## Results

The main goal of this work is to study how the strength of intermolecular interactions affects melt memory during the recrystallization of semicrystalline polymers using MD simulations. In recent years, and particularly over the past decade, numerous experimental studies have underscored the impact of intermolecular interactions on the melt memory of semicrystalline polymers.^28,29,37^

In this study, we systematically scale the intermolecular Lennard-Jones (LJ) interaction parameter (ε) within a simplified United Atom (UA) model of a linear *n*-alkane chain to represent a controlled variation in interchain cohesion strength. We emphasize that this model describes a polyethylene-like system and does not explicitly account for directional interactions such as hydrogen bonding or electrostatic dipole–dipole forces. Instead, the scaling parameter λ provides a physically, albeit chemically simplified, representation of enhanced intermolecular attraction. In chemical terms, increasing the interaction strength mimics the progressive reinforcement of interchain cohesion that accompanies the introduction of polar or hydrogen-bonding functionality along the polymer backbone, as reflected in the higher Hildebrand solubility parameters of the corresponding polymer families. This approach enables a controlled investigation of the emergent melt memory as a function of the interchain cohesion, while maintaining computational efficiency and structural simplicity.

In particular, nonbonded interactions have been modeled using the Lennard-Jones potential in the following form:

1 $$u\left(r_{ij}\right)=4\lambda \varepsilon_{ij}^{0}\left[{\left(\frac{\sigma_{ij}}{r_{ij}}\right)}^{12}-{\left(\frac{\sigma_{ij}}{r_{ij}}\right)}^{6}\right]$$

where ε^0^ = 0.3056 kJ/mol. The scaling factor λ in eq 1 directly modulates the strength of nonbonded interactions between monomers. In this work, we consider λ = 1.0, 1.125, and 1.25; an additional weaker-interaction case (λ = 0.75) was simulated solely for the solubility-parameter comparison in Figure 7C. Similar strategies have been employed in coarse-grained and united-atom simulation studies to approximate the effect of increased intermolecular cohesion without introducing explicit directional potentials.^38–42^ For instance, systematic scaling of the Lennard-Jones ε parameter has been used to capture enhanced effective interactions in polymer-based systems, such as polymer–nanoparticle composites, while avoiding the computational cost associated with more chemically detailed models.^43^ The parameter set and the details of the adopted force field are reported in the Supporting Information Section S1, while preparation of simulated systems is described in the Supporting Information Section S2.

As a first step, we induced crystallization by fast cooling the isotropic melt at a constant cooling rate of 22 K·ns^–1^. For the system with the strongest intermolecular attraction (λ = 1.25), the temperature was decreased from 500 to 280 K. The bulk mass density over the entire temperature range is reported in Figure SI-1A of the Supporting Information. As shown, a sharp increase in density was observed, from approximately 860 kg/m^3^ in the amorphous state to approximately 930 kg/m^3^ in the semicrystalline phase. This abrupt transition (typical of a first-order liquid–solid transition) occurs over a narrow temperature range of 2 K, allowing us to determine the crystallization temperature (*T*_*c*_) to be between 303 and 304 K (see Figure SI-1B).

The obtained semicrystalline morphology was used as the starting point to examine how the strength of intermolecular interactions affects the melting temperature (*T*_*m*_). Figure 2 shows the temperature-dependent evolution of the bulk mass density for the three λ scaling factor values. As illustrated, an increase in λ from 1 to 1.25 leads to a systematic shift of *T*_*m*_ toward higher temperatures, indicating that stronger intermolecular attractions stabilize the crystalline phase; thus higher temperatures are needed to melt the polymer into the isotropic state. While the overall shape of the density profiles remains similar across the three cases, *T*_*m*_ increases from approximately 340 to 380 K as λ is increased from 1 (green dots) to 1.25 (blue dots). In Table 1, *T*_*c*_ and *T*_*m*_ values are reported as functions of the scaling factor.

**Figure 2:**
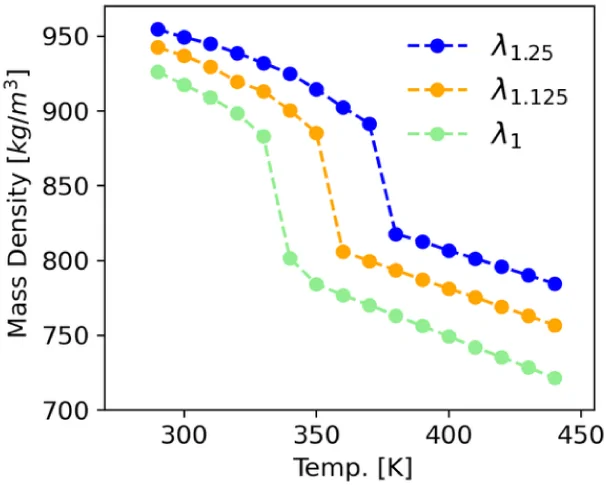
Mass density as a function of temperature for systems with different interaction strengths, λ. Each curve was obtained by stepwise heating of a semicrystalline sample from 290 to 440 K in 10 K increments, with 10 ns of equilibration at each step in the NPT ensemble (*P* = 1 atm).

**Table 1:** Crystallization (*T_c_*) and Melting (*T_m_*) Temperatures of Simulated Systems as a Function of λ

**System**	***T***_***c***_ **(K)**	***T***_***m***_ **(K)**
λ = 1.0	303–304	∼ 340
λ = 1.125	303–304	∼360
λ = 1.25	303–304	∼380

From the plots shown in Figure SI-3, it is evident that melting occurs at approximately 340 K for λ = 1, consistent with the inflection point observed in Figure 2 (green symbols). At temperatures above 340 K, the system is in the melt phase, whereas below this threshold it remains partially unmolten, retaining residual semicrystalline order that progressively diminishes as the temperature approaches *T*_*m*_. A similar behavior is observed for λ = 1.125 and 1.25, as reported in Figures SI-4 and SI-5.

In this context, self-nucleation (*Domain II*) can originate either from surviving crystal fragments in the melt (*Domain IIb* or the self-seeding *Domain*) or from residual chain orientation that persists without true crystalline order (*Domain IIa* or the melt memory *Domain*). In the latter case, the partial alignment of polymer segments within a *nonisotropic* melt (*Domain IIa*) enhances nucleation compared with a fully relaxed, *isotropic* melt (with random coils), leading to melt memory. The *nonisotropic* melt retains structural traces of the original semicrystalline state.^7^ When this residual orientation is fully removed by sufficient heating above the melting temperature, the melt becomes isotropic and corresponds to *Domain I* (the melting domain), depending on the strength of intermolecular interactions.

Melt memory is likely a local phenomenon resulting from interactions among polymer chain segments, which lead to localized regions of enhanced chain packing. To reveal the structural signatures of melt memory, we examined the spatial distribution of local density within the simulated systems. For each representative equilibrium conformation obtained during the heating procedure, three-dimensional density maps, ρ­(*x*,y,z), were generated by subdividing the simulation box into cubic subcells of ∼1.0 nm per side, i.e., about half the radius of gyration of a polymer chain in the melt. The local mass density was calculated for each subcell and renormalized by the average mass density of the perfect crystal, and the resulting values were compiled into histograms to describe their overall distribution. A cutoff of ρ/ρ_0_ = 0.9 was used to identify regions of enhanced local packing, where ρ_0_ is the mass density of the reference perfect crystal (full *trans* chain conformation) simulated at the corresponding λ (Table S1, SI Section S4). The physical basis for this choice is 2-fold. First, as demonstrated in Figures SI-2A and SI-2B, densities above this threshold correspond exclusively to the perfect crystal distribution and are entirely absent in the isotropic melt. Second, and independently, we computed the trans fraction as a function of local density, mapping chain conformational order directly onto the local density. As shown in Figures SI-2A and SI-2B, regions above the threshold in the perfect crystal exhibit a trans fraction consistently near unity (∼1.0), reflecting the extended all-trans chain conformation characteristic of the crystalline lattice. In contrast, no region in the isotropic melt reaches this density range, and the trans fraction remains uniformly low (*∼*0.50–0.65). The convergence of these two independent descriptors, local packing density and local chain conformation, confirms that ρ/ρ_0_ = 0.9 is a reasonable boundary between locally ordered and less-ordered regions. These high-density portions of the melt were therefore interpreted as residual, locally ordered regions that may serve as structural fingerprints of melt memory. Numerical procedures to obtain the density map are reported in Section S5 of the Supporting Information.

For the intermolecular interaction strength level, λ = 1, the plots in Figure SI-6 provide a global view indicating that regions with local density above the defined threshold are present in the unmolten state (from 290 to 330 K). As the system approaches the *T*_*m*_ of the model (∼340 K, see Figure 2), the high-density regions disappear. The trend is clearly visible in the sequence of distributions from 340 to 440 K, see Figure SI-6. In particular, the absence of high-density regions indicates a lack of locally enhanced chain-segment packing and, thus, the absence of structural features possibly associated with melt memory or *Domain IIa*. This behavior is consistent with experimental findings for polyethylene, which typically exhibits only *Domain IIb* or self-seeding and no *Domain IIa*.^10,31^

By increasing the interaction parameter via the scaling factor λ, the attractive forces between atomic sites are effectively strengthened, which not only makes the crystalline phase more resistant to melting (see Figure 2), but also promotes melt memory. These λ values examined in this work span a range of physically distinct behaviors: λ = 1.0 represents the reference case in which no melt memory is observed, λ = 1.25 represents the upper bound of the range explored, and λ *=* 1.125 is the intermediate case that is critical for establishing whether the emergence of melt memory is gradual or threshold-like. As shown in Figure 3 for λ = 1.25 and in Figure SI-7 for λ = 1.125, a residual population of high-density regions above the defined threshold persists in the molten state at temperatures above *T*_*m*_ in both cases, while it is entirely absent for λ = 1.0. More specifically, high-density regions disappear only at a temperature well above *T*_*m*_, at *T* > 410 K in the case of λ = 1.25. Crucially, the extent of this above-threshold population decreases progressively from λ = 1.25 to λ = 1.125, confirming that the phenomenon emerges gradually and continuously with increasing cohesion strength, rather than appearing abruptly at a critical interaction parameter value.

**Figure 3:**
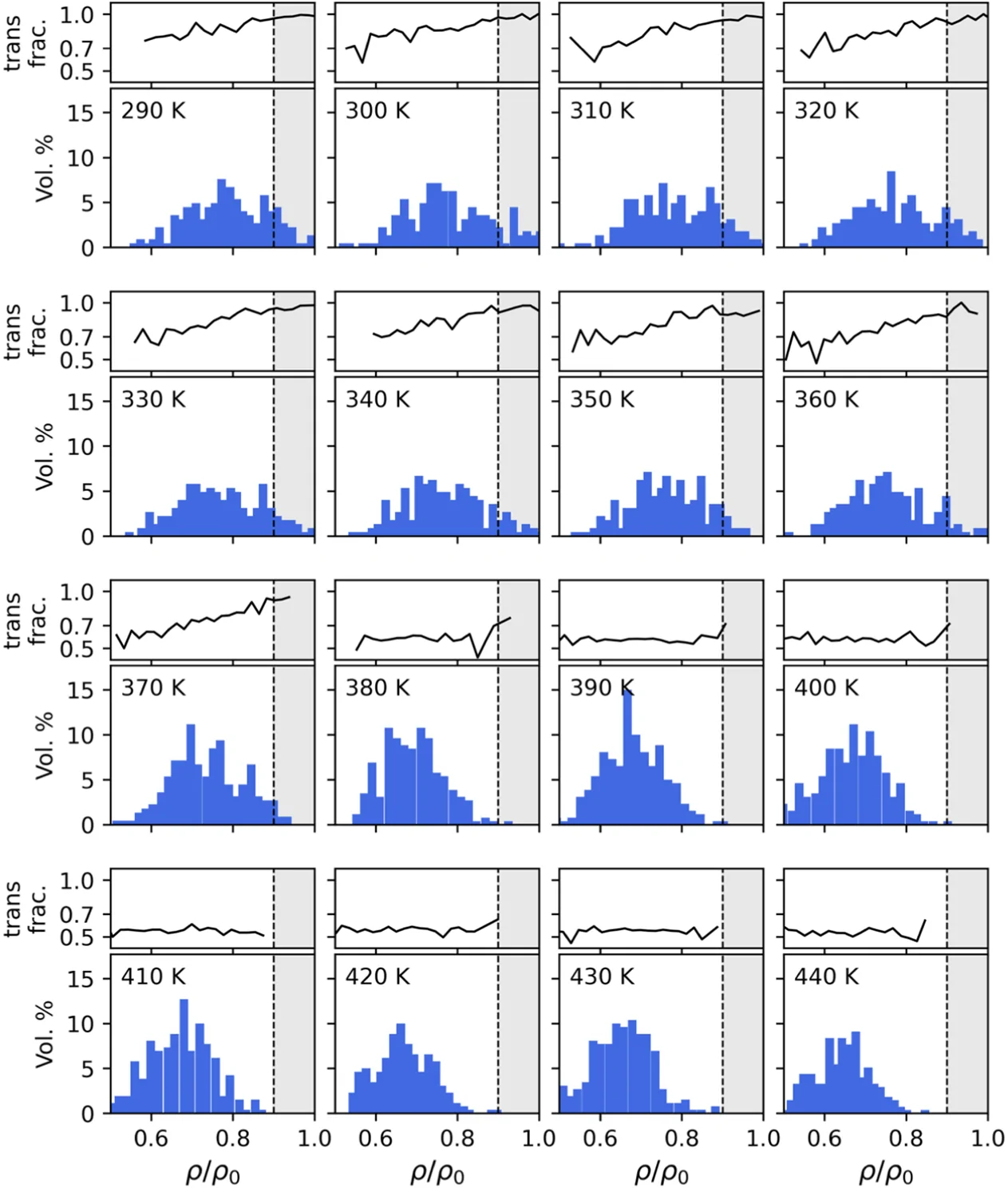
Distribution of local atomic density (ρ/ρ_0_, blue histograms) and the trans fraction (upper panels) as a function of temperature (290–440 K) for an interaction strength of λ_1.25_. The vertical dashed line marks the cutoff at ρ/ρ_0_ = 0.9, corresponding to 90% of the reference ideal crystal density (ρ_0_) for infinite linear chains in the trans-planar conformation simulated at the corresponding λ (Table S1, SI Section S4). For each temperature, the density distribution (bottom panel) is paired with its corresponding trans-fraction distribution (top panel), allowing direct comparison between local structural order and chain conformational state across the explored thermal range.

A clear representation of the temperature dependence of the high-density regions is provided by density iso-surfaces shown in Figure 4A. Conformations at four increasing temperatures, ranging from 290 to 440 K, and λ = 1.25, are displayed, where chain segments within local high-density regions are highlighted in red and the remaining chains in gray. For each snapshot, the corresponding iso-surface represents spatial regions with densities above the threshold indicated in Figure 3 (black dashed line). At 290 and 320 K, corresponding to the semicrystalline state, high-density regions coincide with well-ordered, tightly packed chain segments. At 320 K, disordered chain portions of the melt vanish from the iso-surface, leaving only ordered regions. At the melting temperature (*T*_*m*_ = 380 K), the system is molten, but residual high-density regions persist. These regions progressively diminish as temperature increases and completely disappear at 440 K, as shown in the sequence of histograms in Figure 3. In Figure 4B, eight representative atomistic visualizations of the high-density regions identified in the melt are depicted. These images show the atomic coordinates of chain segments from different molecules, rendered in different colors to facilitate inspection of local packing. The snapshots demonstrate that these dense regions are not remnants of incompletely molten crystals. Indeed, the high-density regions do not exhibit the regular, long-range chain alignment expected for residual crystallites. Rather, they display heterogeneous local organization: short extended chain portions coexist with folded conformations and locally optimized packing motifs, without the long-range translational or orientational order characteristic of crystalline regions. A comparable trend is also observed for the intermediate case λ = 1.125, as shown in Figure SI-7. This behavior contrasts with that found for λ = 1, where such regions vanish at *T*_*m*_ (∼340 K).

**Figure 4:**
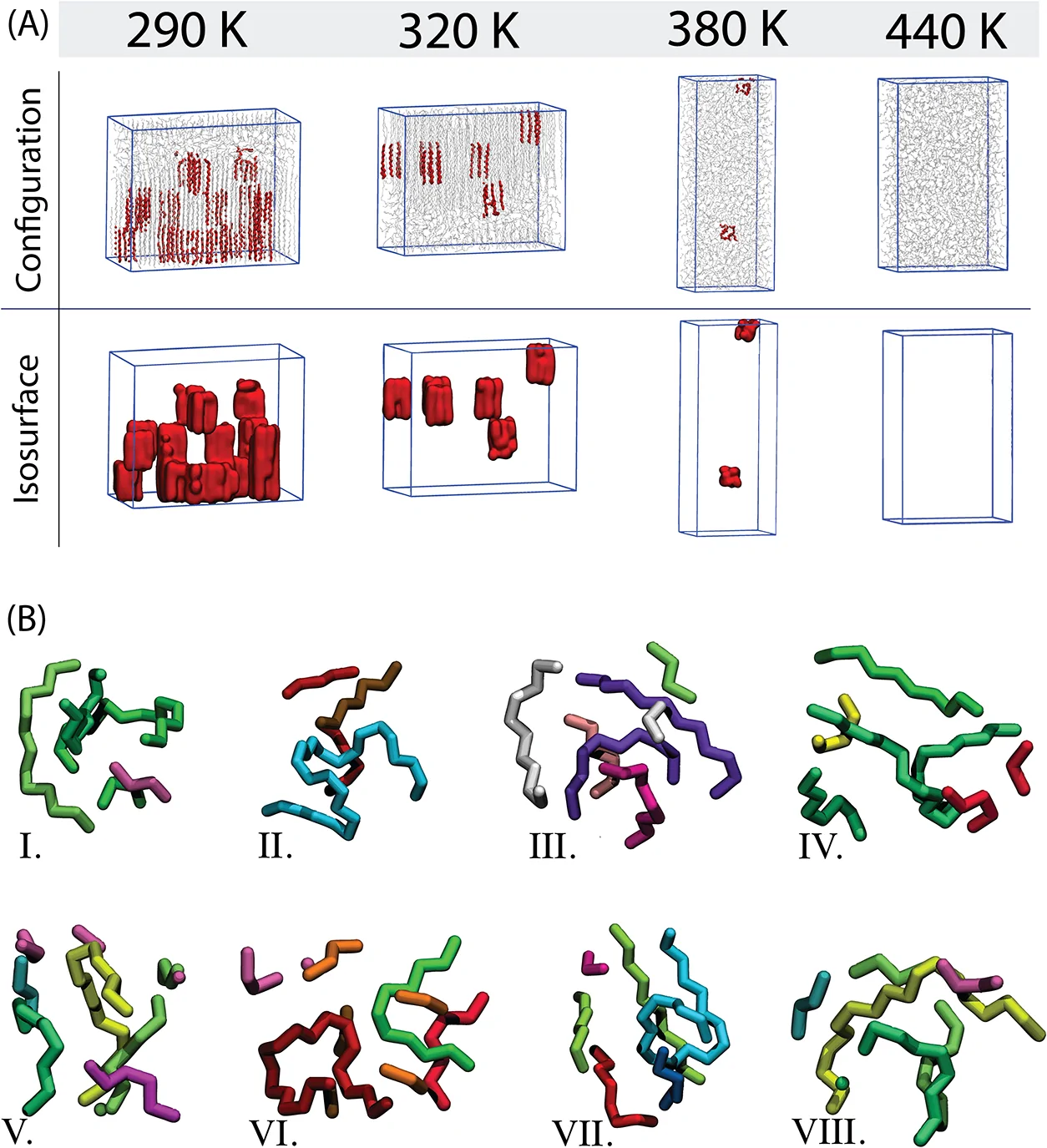
(A) snapshots of simulated systems (λ_1.25_) at four temperatures, from 290 to 440 K, spanning the semicrystalline state and the melt above *T_m_* = 380 K. For each conformation, the corresponding density iso-surface is shown, highlighting regions with local density above the threshold defined in Figure 3 (red). Lower-density regions are omitted for clarity, while segment chains are displayed in the snapshots. (B) representative atomistic views of high-density regions in the melt. Chain segments belonging to different molecules are shown in different colors. The snapshots reveal local packing heterogeneity, including short extended segments, folded chain portions, and locally compact arrangements, but no long-range crystalline order.

Let us now examine how the local density distribution evolves during melting at *T*_*m*_ (380 K) for the system with λ = 1.25. We focus on two representative distributions shown in Figure 5. The first, labeled as (1), corresponds to a state just before melting, where the system remains semicrystalline, and the mass density is still high (Figure 5A and 5C), while the second distribution, labeled as (2), represents the system after it has fully transitioned to the melt (Figure 5A and 5C). The transition to the melt is monitored by tracking the bulk mass density, which decreases abruptly around 3–4 ns, as shown in Figure 5A. A complementary indicator of this transition is the variation of the *trans* conformational fraction of the chains. In the crystal state, chain segments adopt extended all-*trans* conformations within the orthorhombic unit cell. Upon melting, as chains relax toward random coil conformations, the *trans* fraction decreases significantly. Panel B of Figure 5 reports the trans fraction throughout the heating process. Below *T*_*m*_, the fraction of all-*trans* conformations remains high (∼0.9 – 0.8), while a sharp drop is observed at 380 K (*T*_*m*_), marking the onset of the molten state.

**Figure 5:**
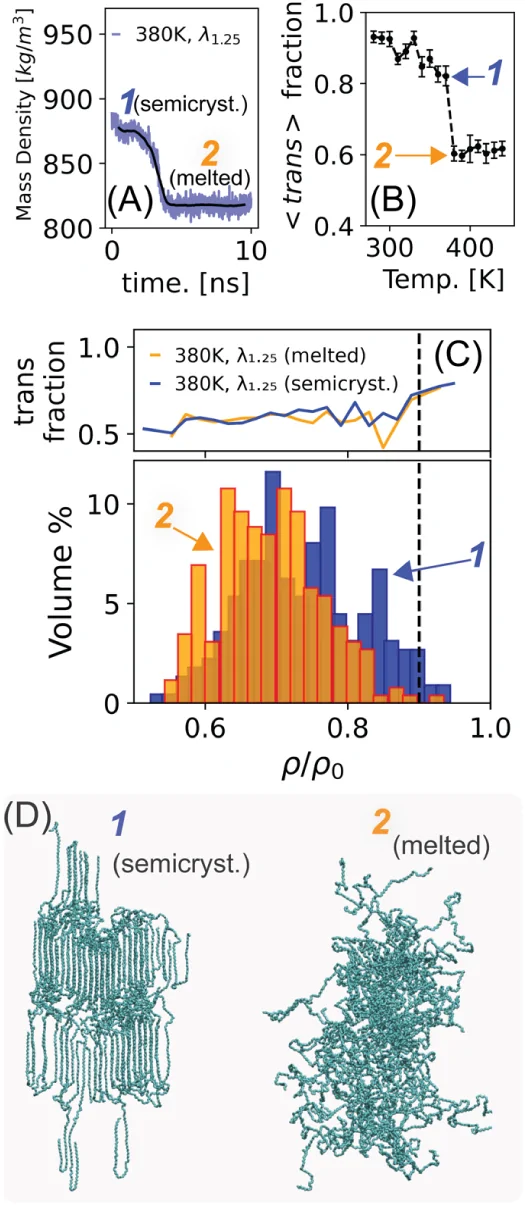
(A) Time evolution of the mass density at *T_m_* for the system with a scaling factor λ_1.25_. The semicrystalline and melt states are indicated as (1) and (2), respectively. (B) Fraction of all-trans conformation of chains as a function of the temperature, along the melting process. Labels (1) and (2) indicate, as in panel A, the semicrystalline and melt states, respectively. (C) Distributions of the local atomic densities and trans fractions calculated at *T_m_* = 380 K for the semicrystalline state blue bars and the melt state orange bars. The vertical dashed line marks the cutoff at ρ/ρ_0_ = 0.9. (D) representative snapshots of simulated systems in semicrystalline and melt states at *T_m_* temperature.

As previously discussed, residual chain order is expected in the melt state when melt memory is present and should correlate with the presence of local high-density regions. A comparison between the local density distributions before (1) and after melting (2) reveals the persistence of such high-density regions in the molten state; see the bars over the threshold in the histogram reported in Figure 5C. Notably, the distribution corresponding to the melted system at *T*_*m*_, shown in orange, is shifted toward lower densities, as expected for a disordered melt. Representative snapshots of the two states, semicrystalline and molten, are shown in Figure 5D.

As shown in Figure 5B, the average trans fraction measured across the entire system varies significantly between the semicrystalline and molten states, indicating the expected conformational change upon melting. However, a different picture emerges when focusing solely on the polymer chains within the high-density regions. In Figure 6A, we present the *trans* fraction throughout the heating process, including the values for the two representative states at *T*_*m*_ (labeled as 1 and 2), as well as the fraction calculated solely for segments located in regions above the density threshold. Notably, the gold diamond indicates a *trans* fraction of ∼0.7 in the melt, which is considerably higher than the global value of ∼0.6 for the same melt state at 380 K (label 2 in Figure 6A). This result supports the existence of temperature-resistant chain ordering in the melt, consistent with the structural characteristics of *Domain IIa,* which are associated with melt memory. The snapshots shown in panel C of the same figure provide a visual representation of the extended chain segments (in trans–trans sequences), within high-density regions, for both the semicrystalline (1) and the molten state (2). These images illustrate the presence of locally ordered chain conformations that persist even after the material has melted. As further confirmation, we analyzed the *trans* fraction for the same system heated to 400 K, a temperature 20 K above *T*_*m*_, where residual high-density regions are still observed (see the distributions in Figure 6B). Remarkably, the *trans* fraction of chain segments within these regions remains comparable to that observed at *T*_*m*_, as indicated by the gold diamond and gray triangle symbols in Figure 6A. Snapshots in Figure 6C reveal that extended segments with long trans sequences are present in the polymer chains located within high-density regions at 400 K, supporting the presence of persistent local chain ordering in the melt.

**Figure 6:**
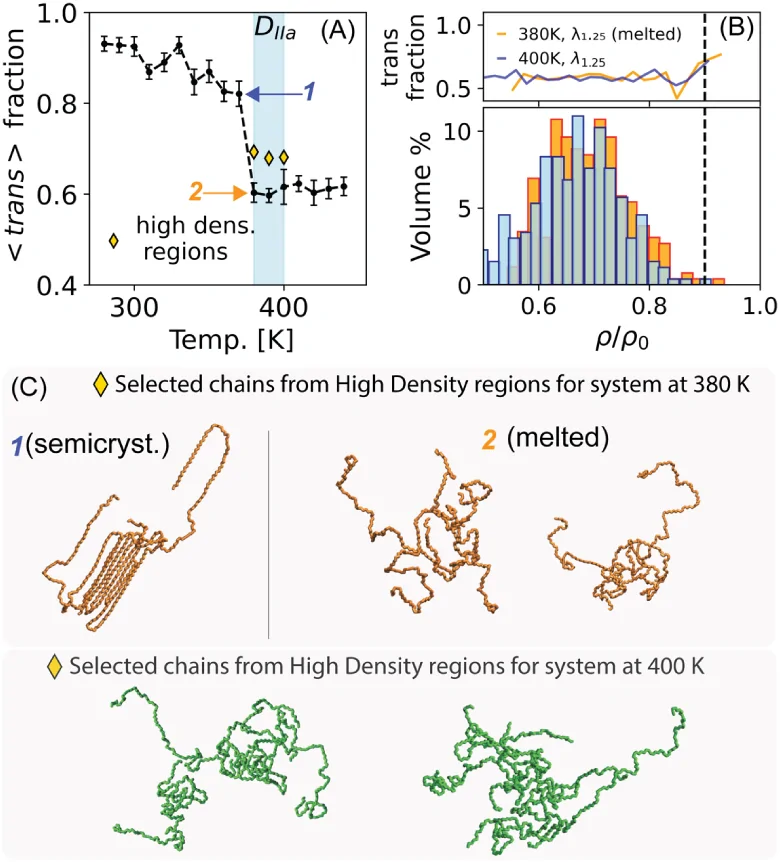
(A) Fraction of all-trans conformation of model chains throughout the heating process. Labels (1) and (2) indicate the semicrystalline and melt states, respectively. The gold diamond marks the fraction calculated for the high-density local regions of the melt state at *T_m_* = 380 K, while the gray triangle indicates the same quantity calculated at 400 K. (B) Distributions of the local atomic densities and the trans fractions calculated at *T_m_* = 380 K for the melt state (orange bars) and for the melt state at 400 K (light-blue bars). The vertical dashed line marks the cutoff at ρ/ρ_0_ = 0.9. (C) Representative snapshots of the simulated system in semicrystalline and melt states at *T_m_* temperatures indicated with the gold diamond, while the gray triangle indicates the chain conformations in the melt state at 400 K.

It is noteworthy that the fraction of chains residing in high-density regions remains nearly constant (approximately 10%) in the melt state at both temperatures: 380 and 400 K (Figure 7A). Furthermore, as shown in Figure 7B, nearly 50% of the chains in high-density regions in the molten state originate from the high-density areas present at 280 K, indicating a degree of chain-order persistence across the first-order phase transition.

**Figure 7:**
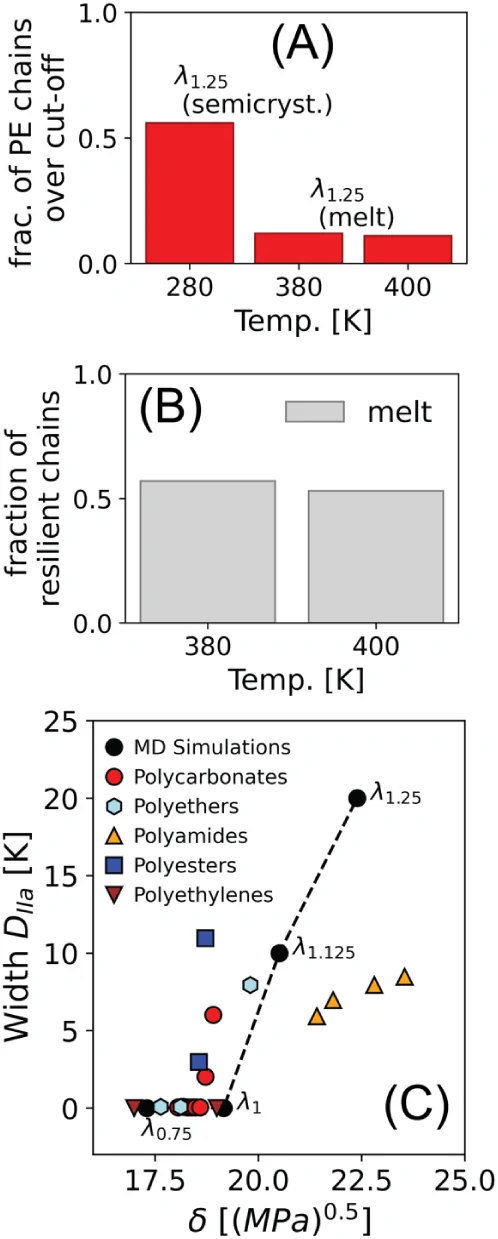
(A) fraction of model chains located in high-density regions (above the density cutoff) as a function of temperature. (B) fraction of chains in the molten state that originally belonged to high-density regions of the semicrystalline sample at 280 K. (C) The width of *Domain IIa* increases with the solubility parameter (δ) for various polymers and MD simulations. MD data for increasing interaction strengths (λ = 0.75; 1.0; 1.125; 1.25) are shown with black circles connected by a dashed black line. It should be noted that the MD-derived solubility parameters are calculated from a polyethylene-like model with scaled LJ cohesion and are not expected to quantitatively reproduce the δ values of specific experimental polymers. The comparison with experimental data is therefore intended to illustrate qualitative trends only rather than to claim quantitative agreement. Details of the solubility parameter calculation from MD simulations are reported in Supporting Information Section S6. Experimental data were taken from ref^28^.

Panel C of Figure 7 highlights a direct link between melt memory and polymer cohesion. The width of *Domain IIa*, the temperature range over which melt memory persists, correlates positively with the solubility parameter (δ) across a variety of polymers,^28^ including polycarbonates, polyethers, polyamides, polyesters, polyethylenes,^44^ as well as MD simulations (black circles). Importantly, the three λ values produce a continuously increasing solubility parameter δ, confirming that the LJ scaling generates a progression in cohesive energy density. The dashed line connecting MD data for increasing interaction strengths (λ = 0.75; 1; 1.125; 1.25) reinforces this trend, showing that above a certain threshold, stronger intermolecular interactions (λ > 1) lead to a broader melt memory *Domain IIa*. These findings suggest that tuning the strength of intermolecular interactions offers a general strategy for controlling melt memory behavior across different polymer systems. For example, *Domain IIa* increases with polyamide cohesive interactions (orange upper triangle). Conversely, *Domain IIa* is absent in the polymer model when the interaction strength is reduced (λ ≤ 1). As λ increases and intermolecular attractions strengthen, features linked to *Domain IIa* gradually appear, consistent with experimental observations.^28,29,45,46^

Heating the system beyond the critical temperature required to reach *Domain* *I* results in a relaxed, isotropic melt, in which polymer chains adopt random-coil conformations. As a result, high-density regions as well as melt memory disappear, as shown in the distribution at 440 K (Figure 8A). When the isotropic melt is subsequently fast-quenched to a temperature *T* = 370 K < *T*_*m*_, no local regions of high packing or high density are observed, indicating that chain order inherited from the semicrystalline phase has been lost. This is clearly illustrated in Figure 8B, which compares the local density distributions of the system heated from the semicrystalline state (blue bars) and that of the memory-erased melt cooled to the same temperature (green bars).

**Figure 8:**
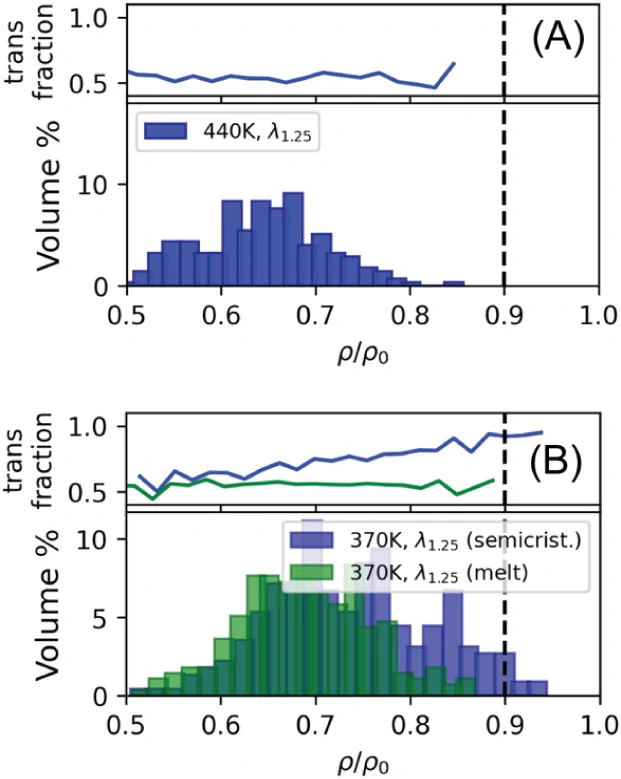
Distribution of local atomic densities and trans fractions calculated at (A) *T* = 440 K for the melt state, and (B) *T* = 370 K < *T_m_*, comparing the semicrystalline state (blue bars) and the melt state (green bars). For each density distribution, the corresponding trans-fraction distribution is reported in the upper panel, allowing direct comparison between local structural order and chain conformational state. The vertical dashed line marks the cutoff at ρ/ρ_0_ = 0.9, corresponding to 90% of the ideal crystal density for infinite linear chains in the trans-planar conformation. Systems were simulated with λ_1.25_.

It is well established that self-nuclei produced by melt memory are highly effective at increasing nucleation density when the sample is cooled from *Domain IIa* (thereby significantly increasing the crystallization temperature; see Figure 1) compared with cooling from an isotropic melt (*Domain I*).^47^ To examine this effect, we selected two systems. The first, representative of *Domain IIa*, corresponds to a molten state at *T* = 380 K with λ = 1.25 which retains regions of residual chain ordering (labeled as state 2 in Figure 5). The second system, referred to as *isotropic,* was prepared by instantaneously quenching the fully relaxed melt at 440 K to *T* = 370 K (λ = 1.250). As previously shown in Figure 8B, this system exhibits no high-density regions or melt memory. Both systems were then cooled down to 300 K at four different constant rates: 1.6, 0.8, 0.4, and 0.2 K·ns^–1^. The resulting evolution of mass density is shown in Figure 9. At the highest cooling rate (1.6 K·ns^–1^, Figure 9A), both systems behave similarly, with crystallization (indicated by a sharp rise in mass density) occurring at around 100 ns. However, at intermediate rates (0.8 and 0.4 K·ns^–1^, Figure 9B and 9C), the system cooled from *Domain IIa* (blue) crystallizes earlier than the isotropic system (red), showing a faster density increase. The most pronounced difference is observed at the slowest cooling rate of 0.2 K·ns^–1^ (Figure 9D), where only the *Domain IIa* system exhibits a crystallization transition within the simulation time frame.

**Figure 9:**
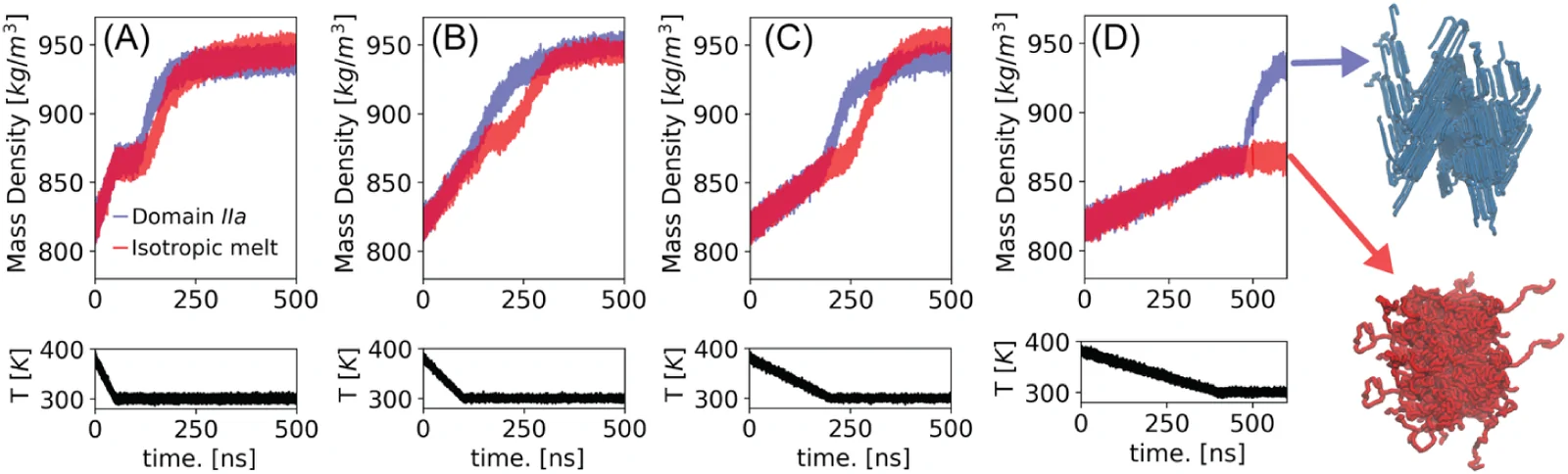
Quenching procedures at different cooling rates: (A) 1.6 K·ns^–1^, (B) 0.8 K·ns^–1^, (C) 0.4 K·ns^–1^, and (D) 0.2 K·ns^–1^. Density evolution is shown in blue for the system initialized from the Domain IIa melt at 380 K, in which melt memory is preserved, and in red for the isotropic system initialized from the fully relaxed melt quenched from 440 to 370 K.

These findings confirm that the *nonisotropic* melt structure, which is difficult to visualize experimentally and believed to stem from residual chain orientation inherited from the crystalline state,^46,48–50^ promotes primary nucleation much more efficiently than an isotropic melt. Notably, this enhanced crystallization effect is captured in MD simulations despite the use of relatively fast cooling rates compared to experimental time scales. The locally ordered, high-density regions identified in this work represent the molecular-scale structural signature of this nonisotropic character: persistent, conformationally biased regions that act as preorganized templates for nucleation. As a counterproof, we selectively disrupted these regions via a dual-thermostat erasure experiment while leaving the global melt state unchanged (Section S8 of Supporting Information). The system, once deprived of its high-density regions, failed to crystallize under the very same slow cooling conditions (cooling rate at 0.2 K·ns^–1^) that produce clear crystallization in the intact *Domain IIa* system, directly demonstrating that the presence of these locally ordered regions is not merely correlated with, but causally responsible for, the accelerated crystallization associated with melt memory.

## Methods

We employ a simplified United Atom (UA) model of linear model chains in which each repeating unit is represented by a single interaction site.^51^ All simulated systems consisted of 60 linear model chains, each containing 150 interaction sites. The starting conformations of dense polymer melt models were obtained by a pre-equilibration procedure using the hybrid particle-field (hPF) approach,^52,53^ following the procedure reported by De Nicola et al.^54,55^ These runs were performed with the OCCAM-MD code.^56^ This procedure enables realistic conformations for amorphous or melt atomistic models that are in good agreement with available experimental data^55,57,58^ Additional details regarding the force-field parameters, procedure, computational details, and the used model are provided in the Supporting Information Sections S1–S4.

## Conclusions

This work provides molecular-level evidence that melt memory in semicrystalline polymers arises from the persistence of local chain order stabilized by intermolecular interactions. By systematically varying the strength of interchain attractions in a simple linear-chain model, we demonstrate that stronger molecular cohesion promotes the formation and thermal stability of localized high-density regions in the melt. Although these regions lack long-range crystalline order, they retain partial conformational alignment inherited from the previous crystalline state and serve as efficient nucleation sites during subsequent recrystallization.

Our molecular dynamics simulations reveal the transition from a fully isotropic melt to a self-nucleating melt and demonstrate that memory retention arises from interaction-stabilized local order rather than from a completely random chain state. This mechanism explains experimental observations showing that polymers with stronger dipolar or hydrogen-bonding interactions exhibit a more persistent melt memory than systems dominated by weaker dispersive interactions.

Beyond elucidating the molecular origin of melt memory, these findings establish a direct link among microscopic intermolecular forces, structural persistence in the melt, and the macroscopic crystallization behavior. This framework recasts melt memory from an empirical phenomenon to a molecularly tunable property, suggesting that melt memory can be programmed or suppressed through chemical design strategies that control interchain cohesion such as comonomer incorporation, backbone functionality, or specific intermolecular interactions. More broadly, these insights provide guiding principles for manipulating polymer crystallization pathways and engineering semicrystalline materials with tailored structures and properties.

## Supplementary Material





## Notes

Video showing recrystallization upon ultrafast quenching from the melt at 380 K (λ = 1.25); chains retaining trans order from the semicrystalline state (yellow) act as self-nuclei, initiating and guiding crystallization; the simulation, performed in the NPT ensemble, shows changes in box shape and volume during recrystallization (MPG)
More details of the simulation procedure, including a description of the United Atom (UA) model (Section S1); polymer melt setup and equilibration (Section S2); crystallization of the bulk polymer (Section S3); perfect crystal details (Section S4); calculation of local mass density and trans fraction (Section S5); calculation of the Hildebrand parameter (δ) (Section S6); additional analyses (Section S7) and selective erasure of high-density regions (Section S8) (PDF)
