# Supplementary material for: How Polymer Chains Remember Their Crystalline Past: Simulations Uncover the Molecular Origin of Melt Memory
Source: J Am Chem Soc. 2026 Aug 14;148(33):36262–71. doi: 10.1021/jacs.6c13790 (PMC13523754; doi:10.1021/jacs.6c13790)
Supplement: Supplementary file 1 [file ja6c13790_si_002.pdf]

Supporting Information

How Polymer Chains Remember Their Crystalline Past: Simulations Uncover the Molecular Origin of Melt Memory

Antonio De Nicola,*[1,2] Alejandro J. Müller,*[3,4] Dario Cavallo,[5] Giuseppe Milano[1]

[1] Department of Chemical, Materials and Production Engineering, University of Naples Federico II, Piazzale V. Tecchio, 80, 80125 Napoli, Italy

[2] Research Center for Organic Electronics (ROEL), Yamagata University, Jonan 4-3-16, Yonezawa 992-8510, Japan

[3] POLYMAT and Department of Polymers and Advanced Materials: Physics, Chemistry and Technology, Faculty of Chemistry, University of the Basque Country UPV/EHU, Paseo Manuel de Lardizabal 3, 20018, Donostia-San Sebastián, Spain

[4] IKERBASQUE, Basque Foundation for Science, Bilbao, 48009, Spain

[5]University of Genoa, Department of Chemistry and Industrial Chemistry, Via Dodecaneso 31, 16146, Genoa (Italy)

Table of Contents

TABLE OF CONTENTS **2**

S1. UNITED ATOM (UA) MODEL **3**

S2. POLYMER MELT SET-UP AND EQUILIBRATION **5**

S3. CRYSTALLIZATION OF BULK POLYMER **6**

S4. PERFECT CRYSTAL **8**

S5. CALCULATION OF LOCAL MASS DENSITY AND TRANS FRACTION **10**

S6. CALCULATION OF HILDEBRAND PARAMETER (δ) **12**

S7. ADDITIONAL ANALYSES **14**

S8. SELECTIVE ERASURE OF HIGH-DENSITY REGIONS **19**

REFERENCES **23**

S1. United Atom (UA) Model

The homopolymer system composed of linear chains is modeled by using a United Atom (UA) representation, in which hydrogen atoms are treated implicitly. This approach maintains most of the conformational degree of freedom of a fully atomistic model while significantly reducing computational expense. Among commonly used UA force fields for alkane chains are PSY[1] (Paul, Yoon, and Smith) and its modification by Waheed et al.,[2] the Yamamoto model,[3–5] and TraPPE-UA (Transferable Potentials for Phase Equilibria – United Atom).[6] In our study, the linear chains are described with the TraPPE-UA force field, in which each CH_2 or CH_3 group is treated as a single interaction site. TraPPE-UA was chosen for its proven ability to accurately reproduce vapor–liquid coexistence curves (VLCC) for *n*-alkanes, as well as structural, dynamical, and thermodynamic properties of melt phases, including mass density and melting behavior. A schematic representation of a single polymer linear chain in the UA model is shown in Scheme S1.

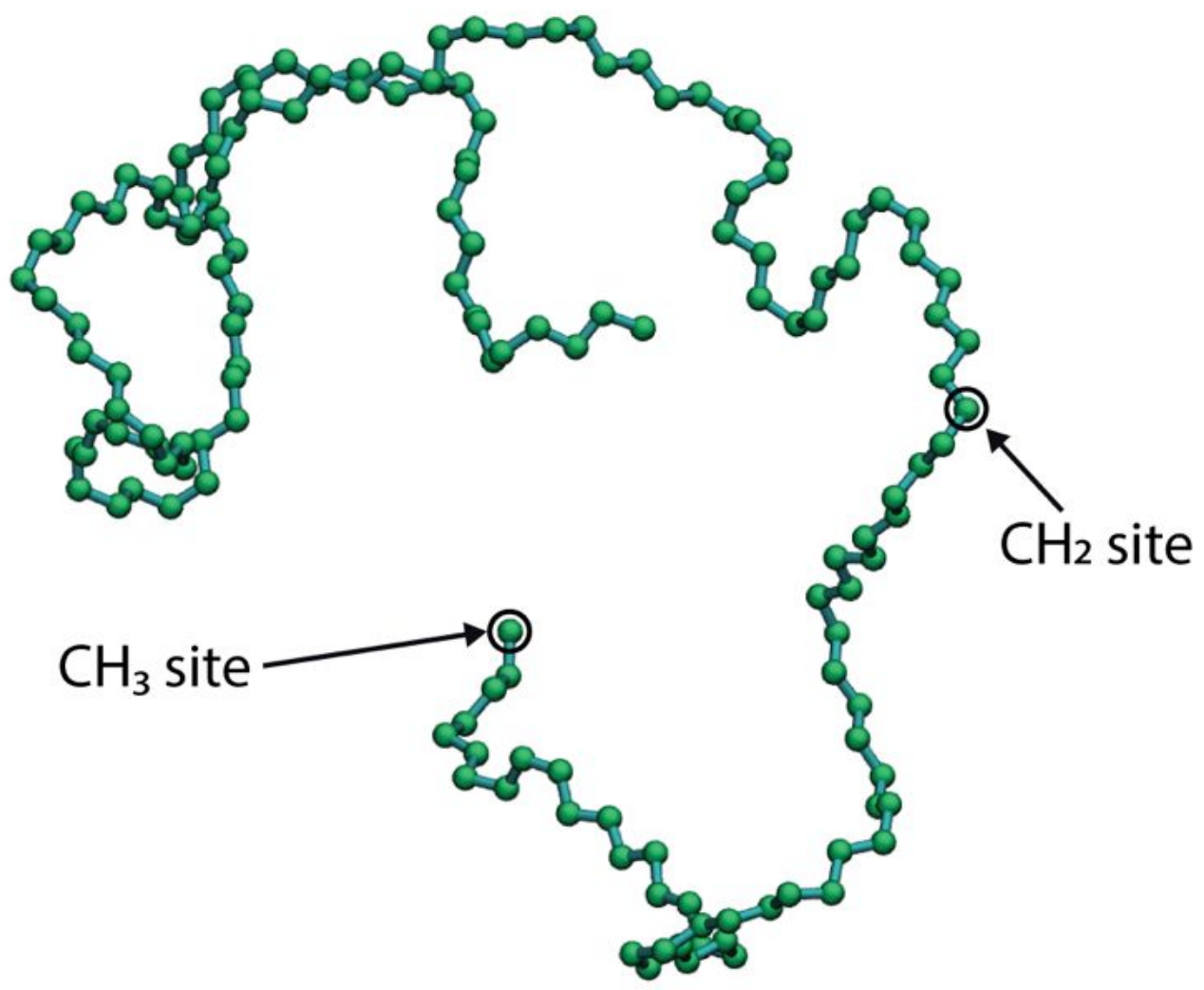

Scheme S1. Schematic representation of a single linear chain. Each bead corresponds to a United Atom interaction site. Terminal beads represent CH_3 groups, whereas intrachain beads correspond to CH_2 groups.

Force-field interactions in molecular simulations are commonly described using effective potential functions that account for both non-bonded and bonded contributions. Non-bonded interactions are typically modeled using the Lennard–Jones (LJ) potential, whereas bonded interactions are represented by harmonic or torsional potentials. The LJ potential for a pair of interaction sites i and j is given by:

$$u(r_{ij}) = 4\epsilon_{ij}\left[\left(\frac{\sigma_{ij}}{r_{ij}}\right)^{12} - \left(\frac{\sigma_{ij}}{r_{ij}}\right)^{6}\right] \quad \text{(S1)}$$

where r_{ij} denotes the inter-site distance, σ_{ij} is the effective size parameter of the interaction sites, and $\epsilon_{ij}$is the depth of the potential well that characterizes the strength of the interaction. The Lennard–Jones parameters are defined as follows: for CH_2, σ_{CH_2} = 0.395 nm and for CH_3, σ_{CH_3} = 0.375 nm. For the scaling parameter $\lambda_{1.25}$ ϵ_{CH_2} = 0.382 $kJmol^{-1}$ and ϵ_{CH_3} = 0.814 $kJmol^{-1}$. The scaling factor λ is applied exclusively to the ϵ_{ij} parameters, while σ_{ij} remain unchanged.

Bonded interactions along the polymer linear chain are described by harmonic potentials. The bond stretching between two consecutive sites is modeled as:

$$E_{bond} = K_b(r - r_0)^2, \quad \text{(S2)}$$

where r_0 is the equilibrium bond length, set to 0.154 nm in this model, and K_b is the bond force constant, with a value of 188.100 $kJmol^{-1}nm^{-2}$.

The bond angle potential for three consecutive sites is expressed as:

$$E_{angle} = \theta_a(\theta - \theta_0)^2, \quad \text{(S3)}$$

with a force constant $K_a = 285.6\ kJmol^{-1}rad^{-2}$. The equilibrium bond angle θ_0 is fixed at 114 deg for the current model. The torsional potential describing four consecutive sites (CHx-CH_2-CH_2-CHx, x=2,3) is represented by a Fourier series expansion:

$$E_{torsion} = C_0 + C_1cos(\phi) + C_2cos^2(\phi) + C_3cos^3(\phi), \quad \text{(S4)}$$

where the coefficients defined as: C_0 = 8.39 (kJ/mol), C_1 = 16.77 (kJ/mol), C_2= 1.13 (kJ/mol), C_3 = -26.29 (kJ/mol).

S2. Polymer Melt Set-up and equilibration

Initial configurations of the systems were generated with 60 linear chains, each composed of 150 united-atom (UA) sites, at a mass density of 0.79 $g\cdot cm^{-3}$, consistent with the experimental density of a low-density homopolymer melt (similar to polyethylene) at 500 K. The simulation box was set as orthorhombic, with periodic boundary conditions (PBCs) applied in all three spatial directions. Pre-equilibration was carried out in the NVT ensemble at 500 K for 150 ns using the hybrid particle-field (hPF) approach,[7,8] following the procedure reported by De Nicola et al.[9,10] These runs were performed with the OCCAM-MD code.[11] Subsequently, a short equilibration phase of 20 ns was performed using standard molecular dynamics (MD) in the NVT ensemble at 500 K. Production runs were conducted in the NPT ensemble at 1 atm using the GROMACS simulation package.[12] Pressure was maintained using the Berendsen barostat (τₚ = 1.0 ps), and temperature was controlled with the velocity-rescaling thermostat (τₜ = 0.5 ps). A time step of 2 *fs* was employed, and Lennard-Jones interactions were truncated at a cut-off distance of 1.4 nm.

S3. Crystallization of Bulk Polymer

Crystallization of bulk homopolymer can be induced by cooling an initially amorphous system at a constant rate. To estimate the crystallization temperature (T_c) of the model employed in this study, we performed five independent simulations on systems consisting of 60 linear chains each, covering the temperature range from 500 K to 280 K. The resulting mass density scan is reported in Figure SI-1A. An abrupt density increase from ~860 kg·m^{-3} (liquid) to ~930 kg·m^{-3} (solid phase) is observed within a narrow temperature interval around 300 K. The analysis of density variations within the temperature range of 303-310 K enables the identification of the minimum temperature at which a characteristic first-order liquid-solid phase transition occurs (Figure SI-1B). Based on these observations, and employing a cooling rate of 22 $K\ ns^{-1}$, the crystallization temperature T_c is estimated to lie within the range of 303-304 K. As crystalline domains develop over time, the orientational order parameter P_2 increases markedly, from approximately 0.3 to 0.7 (Figure SI-1C). P_2 is defined as the ensemble average of the second-order Legendre polynomial:

$$P_2 =< P_2\left(cos\theta_{i,k}\right) >= \left\langle 3 \cos^2 \theta_{j,k} - \frac{1}{2} \right\rangle, \qquad \text{(S5)}$$

where $\theta_{j,k}$ is the angle between the vector formed by sites (i -1) and (i + 1) and the vector formed by the sites (k -1) and (k + 1). The averaging is performed over all such vector pairs and across all chains. P_2 = 0 corresponds to an isotropic state, while P_2 = 1 indicates perfect segmental alignment.

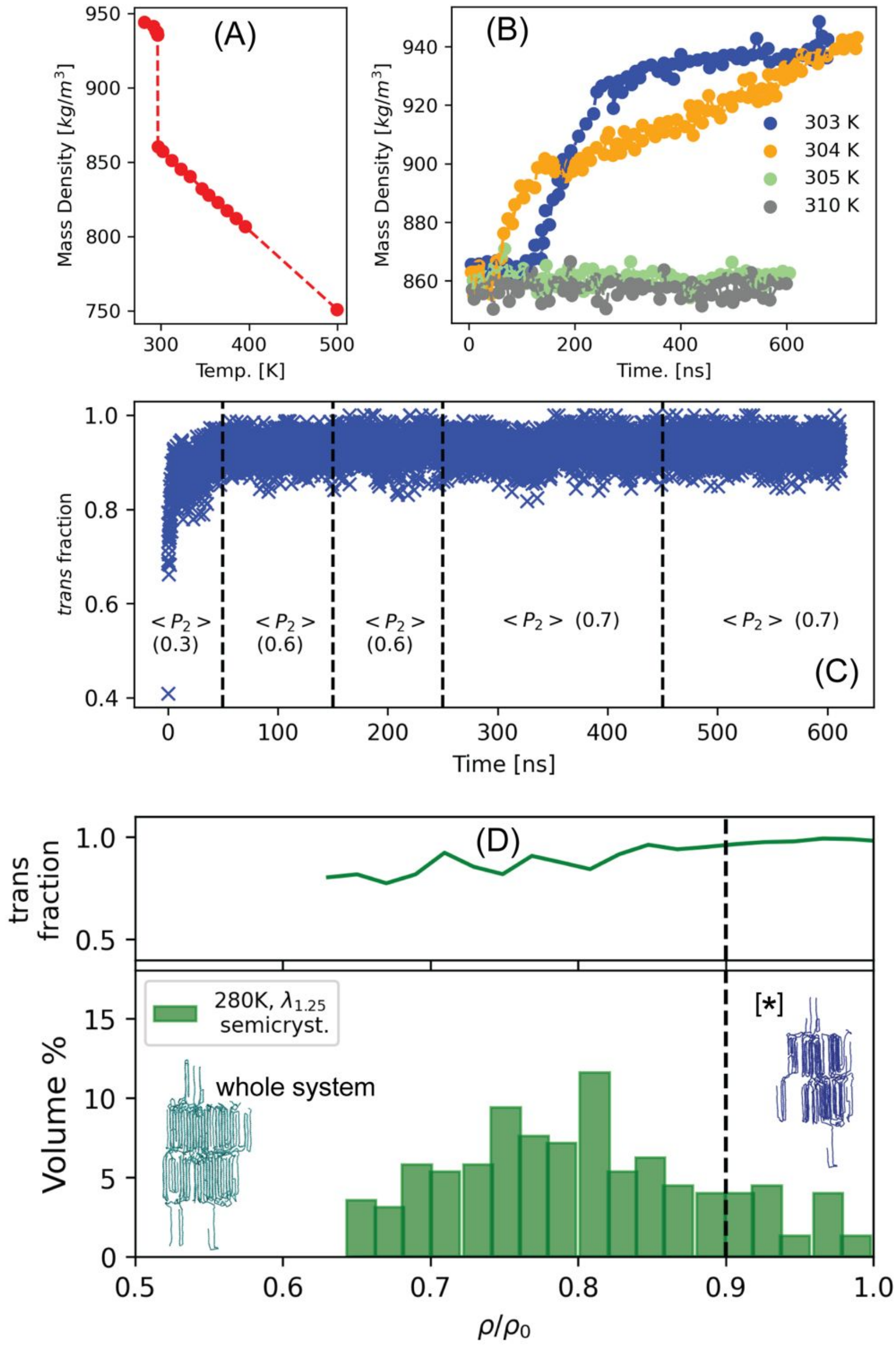

Figure SI-1. (A) Mass density as a function of temperature. Each red point represents a set of 3 independent simulation runs. (B) Mass density as a function of simulation time for four different temperatures: 303, 304, 305, and 310 K. (C) Time evolution of the *trans* fraction. In the same panel, the <P_2> parameter is reported for five-time windows. (D) Histogram of the normalized density ρ/ρ_0 and trans fraction (upper panels) for the semi-crystalline homopolymer sample obtained at 280 K with a scaling factor $\lambda = 1.25$. The histogram has been calculated by averaging the last 20 ns of the production run. The black dashed line at $\rho/\rho_0 = 0.9$ represents the density threshold above which the local density exceeds the reference crystalline density. The background (light gray) shows the full semi-crystalline homopolymer configuration, while chains located in regions above the density cut-off are highlighted in blue and marked with an asterisk on the right side of the panel.

S4. Perfect Crystal

A reference perfect crystal was constructed in the fully trans chain-conformation, representing the ideal packing arrangement. The experimental unit cell[13] of a polyethylene (PE) crystal was used as an initial template to build the system. Specifically, coordinates of the backbone carbon atoms of the PE unit cell were replicated ten times along the x, y, and z directions, resulting in a simulation box containing 50 linear chains with dimensions of $3.92324 \times 2.9339 \times 5.06577,\ nm^3$. In Figure SI-2C,D, snapshots showing the lateral and top views of the crystalline slab are reported, together with the X-ray pattern comparison between the experimental data, measured under similar conditions on a semi-crystalline sample (black line), and the perfect crystal from MD (blue line). The resulting slab was used to compute the trans fraction in semi-crystalline systems. To verify the stability of the reference crystal, molecular dynamics simulations were performed with three different values of the Lennard–Jones energy scaling factor λ. In Table S1, the average mass density, calculated on the last 20 ns of the production run, is reported for each scaling factor λ.

Table S1. Bulk mass density as a function of λ. All the simulations have been performed in NPT with T = 280 K, and P = 1 atm.

**System**	λ	**Mass Density** ($Kg\ m^{-3}$)
Perfect crystal	1.0	921 (0.16)
Perfect crystal	1.125	987 (0.31)
Perfect crystal	1.25	1016.74 (0.24)

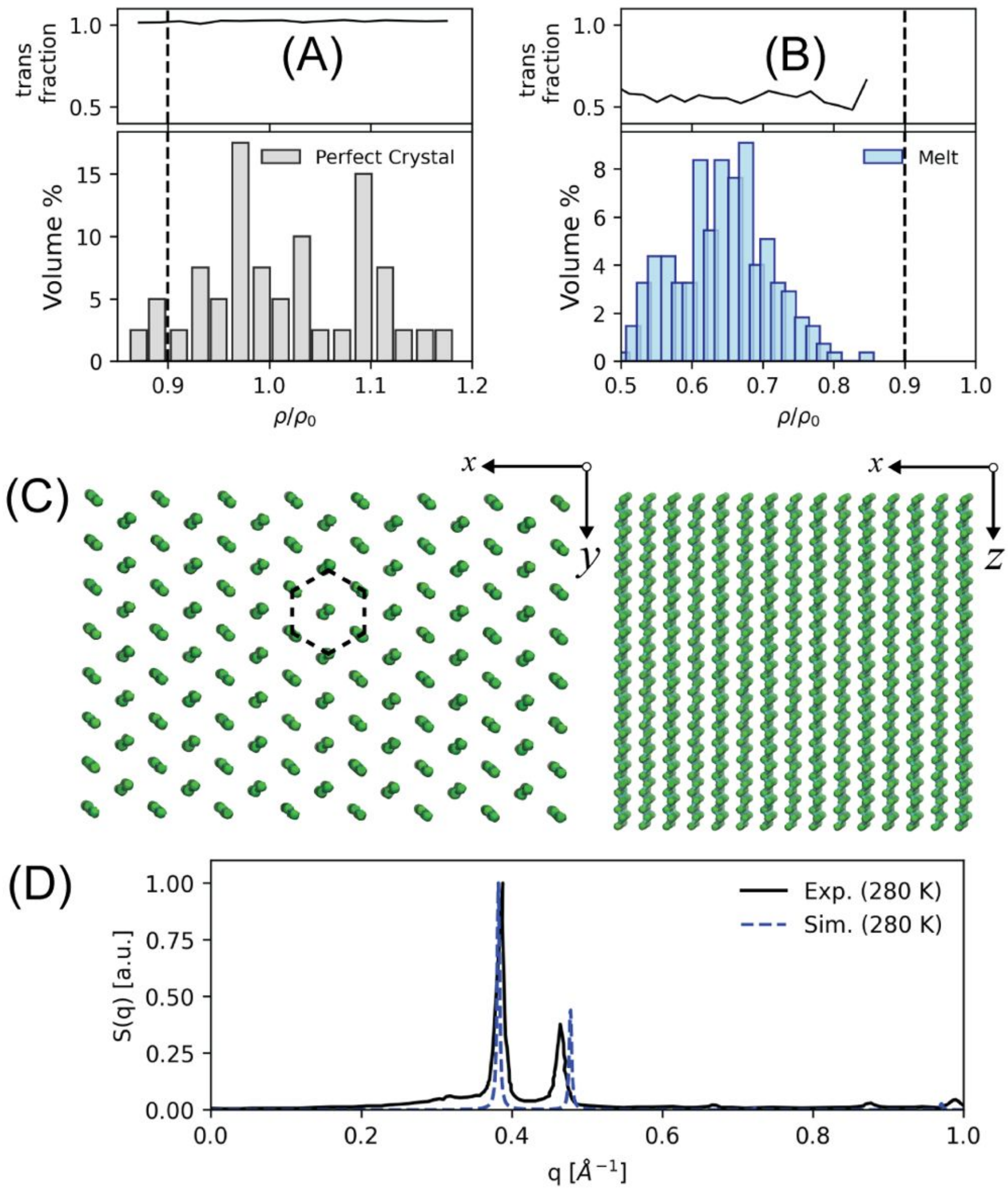

Figure SI-2. Local density distributions (bar plot) and trans fraction (upper panels) calculated on: (A) a perfect crystal and (B) an isotropic homopolymer melt. The definition of the cut-off threshold, represented by the vertical dashed lines in both panels A and B, is reported in Figure 3 of the main text. (C) Two views of a replicated crystal slab, top *xy* plane and lateral *xz* plane. (D) X-Ray pattern from experimental semi-crystalline sample[13] (black line), and calculated from MD simulation at 280 K with a scaling factor $\lambda = 1.25$ (blue line).

S5. Calculation of Local Mass Density and Trans Fraction

To quantify spatial density heterogeneities and identify high-density regions potentially associated with residual chain ordering, we employed a grid-based local density analysis. The simulation box was partitioned into cubic subcells of side length l = 1.0 nm, corresponding to a volume:

$$V_{\mathrm{subcell}} = l^3 = 1.0\ nm^3 \tag{S6}$$

which is approximately one-half of the radius of gyration of a PE chain in the melt state. This choice ensures sufficient resolution while maintaining adequate statistical sampling. For each subcell i, the number of UA sites N_i (CH_2 or CH_3 groups) was counted and converted into a local mass density:

$$\rho_i = \frac{1}{V_{subcell}} \sum_{i=1}^{N_i} m_j \tag{S7}$$

where m_j is the mass of the j-th UA site. The resulting local densities ρ_i were collected over all subcells and simulation frames to build normalized histograms. To facilitate comparison across interaction strengths, densities were normalized with respect to the crystalline density ρ_{cryst} of PE obtained from the model:

$$\rho_i^* = \frac{\rho_i}{\rho_{cryst}} \tag{S8}$$

The applied numerical workflow used to compute ρ_i is listed below:

Numerical Workflow

- Grid construction
  - Divide the simulation box into cubic subcells of side 1.0 nm.
  - Apply periodic boundary conditions to account for subcells spanning box edges.
- Mass density calculation
  - Count UA sites (CH_2/CH_3 groups) in each subcell.
  - Compute local density using eq S7.
- Normalization

Normalize each subcell density using eq S8.

Using the same grid-based approach, we computed the local trans fraction within each subcell. Specifically, dihedral angles ϕ were evaluated along the polymer backbone as:

$$\phi = \angle\left(r_1, r_2, r_3, r_4\right) \qquad \text{(S9)}$$

where r_1, r_2, r_3, r_4 are the positions of four consecutive atoms along a chain, and all are required to lie within the same subcell. Dihedrals that did not satisfy both conditions were excluded from the analysis. A dihedral is classified as *trans* if it satisfies:

$$|\phi - 180°| < \Delta\phi, \ \ (\Delta\phi = 10°) \qquad \text{(S10)}$$

The local trans fraction is then obtained:

$$f_{trans} = \frac{N_{trans}}{N_{total}} \tag{S11}$$

where N_{trans} is the number of dihedrals in the trans state, and N_{total} is the total number of dihedrals considered within the subcell.

S6. Calculation of Hildebrand Parameter (δ)

The Hildebrand solubility parameter (δ) provides a measure of the cohesive energy density of a material and is defined as:[14]

$$\delta = \sqrt{\frac{E_{coh}}{V_m}} \tag{S12}$$

where E_{coh} is the molar cohesive energy ($J \cdot mo^{-1}$) and V_m is the molar volume ($m^3 \cdot mol^{-1}$). In this work, δ has been calculated by following the procedure reported in ref. 14

The cohesive energy E_{coh} was determined from the difference between the total potential energy of the liquid system (polymer melt) and the potential energy of isolated chains. To this end, a single chain was simulated in a large vacuum box to compute the intermolecular energy contributions. The total intermolecular potential U_{inter} is calculated as:[14]

$$U_{inter} = U_{liq,total} - N \cdot U_{intra,mol} \quad \text{(S13)}$$

where:

- $U_{liq,total}$ is the total potential energy of the polymer melt
- $U_{intra,mol}$ is the potential energy of a single polymer chain in vacuum.
- N is the number of chains (60) in the system.

The molar cohesive energy was then calculated as:

$$E_{coh} = -\, U_{inter,per\ mol} \quad \text{(S14)}$$

Positive E_{coh} indicates attractive intermolecular interactions. The molar volume V_m was calculated from the simulation box volume V_{box} and from the number of chains N as:

$$V_m = \frac{V_{box}}{N} \cdot N_A \quad \text{(S15)}$$

where N_A is Avogadro's number. Units were converted from nm^3 to m^3 for consistency in SI units.

S7. Additional Analyses

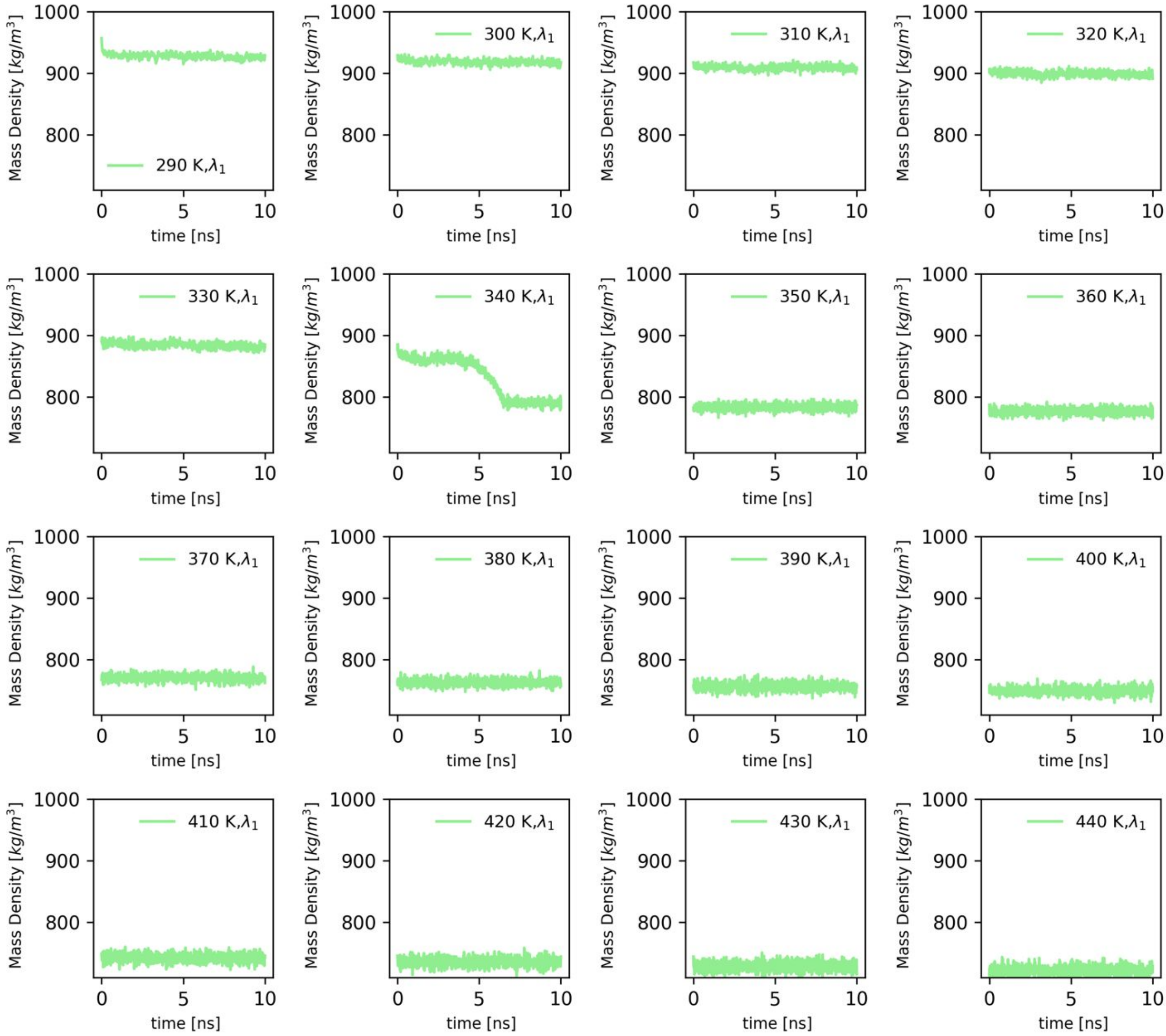

Figure SI-3. Time behavior of Mass Density as a function of heating temperature (reported in each panel). The temperature ranges from 290 to 440 K. The starting configuration at 290K is the semi-crystalline PE bulk. Interaction strength has been set to $\lambda = 1.0$.

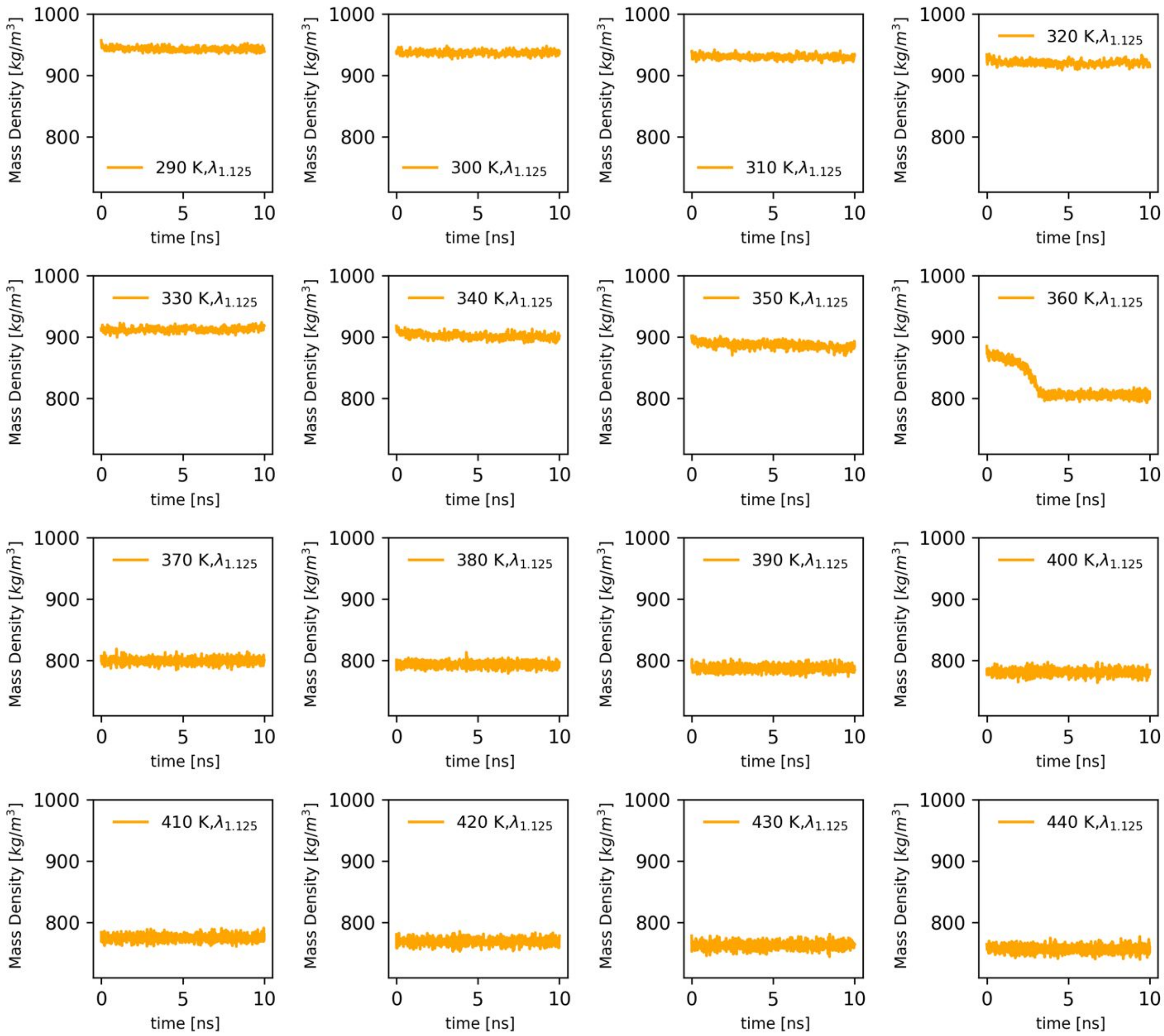

Figure SI-4. Time behavior of Mass-Density as a function of heating temperature (reported in each panel). The temperature ranges from 290 to 440 K. The starting configuration at 290K is the semi-crystalline PE bulk (see Figure 1). Interaction strength has been set to $\lambda = 1.125$.

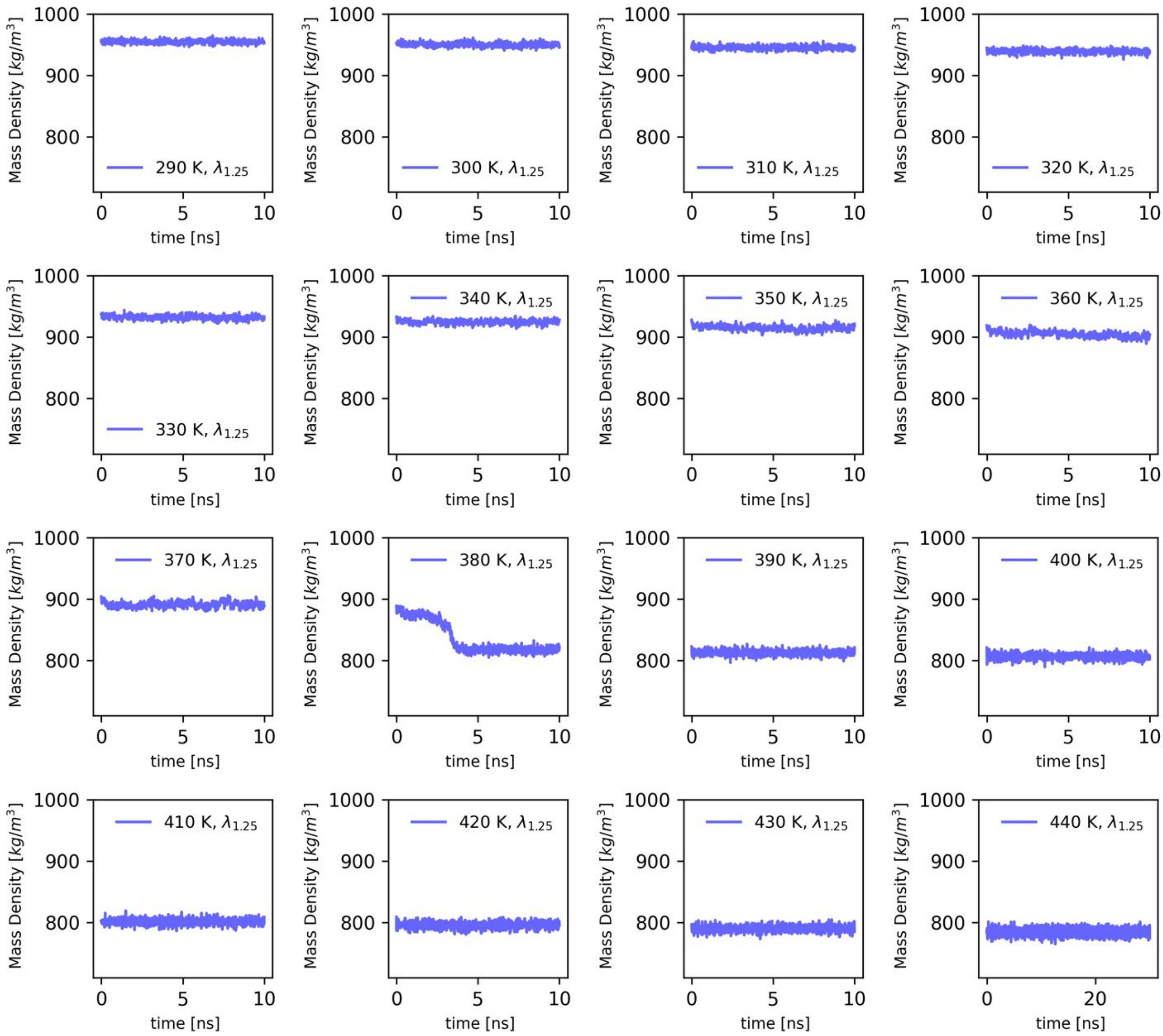

Figure SI-5. Time behavior of Mass-Density as a function of heating temperature (reported in each panel). The temperature ranges from 290 to 440 K. The starting configuration at 290K is the semi-crystalline bulk PE. Interaction strength has been set to $\lambda = 1.25$.

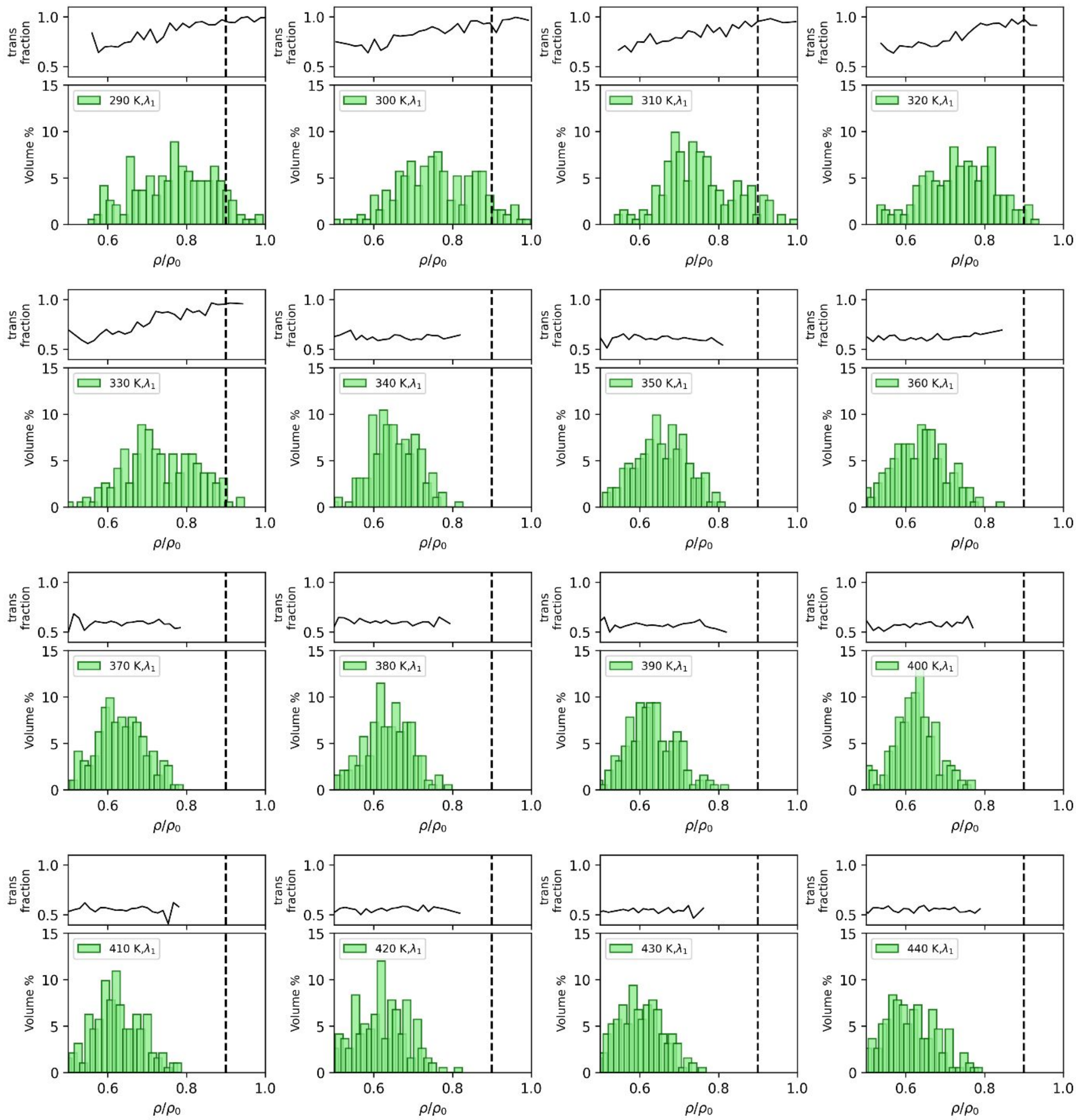

Figure SI-6. Distribution of local atomic density (ρ/ρ_0, green histograms) and the trans fraction (upper panels) as a function of temperature (290-440 K) for an interaction strength $\lambda = 1.0$. The vertical dashed line marks the cutoff at $\rho/\rho_0 = 0.9$, corresponding to 90% of the ideal-crystal density for infinite linear chains in the trans-planar conformation. For each temperature, the density distribution (bottom panel) is paired with its corresponding trans-fraction distribution (top panel), enabling direct comparison between local structural order and chain conformational state across the explored thermal range.

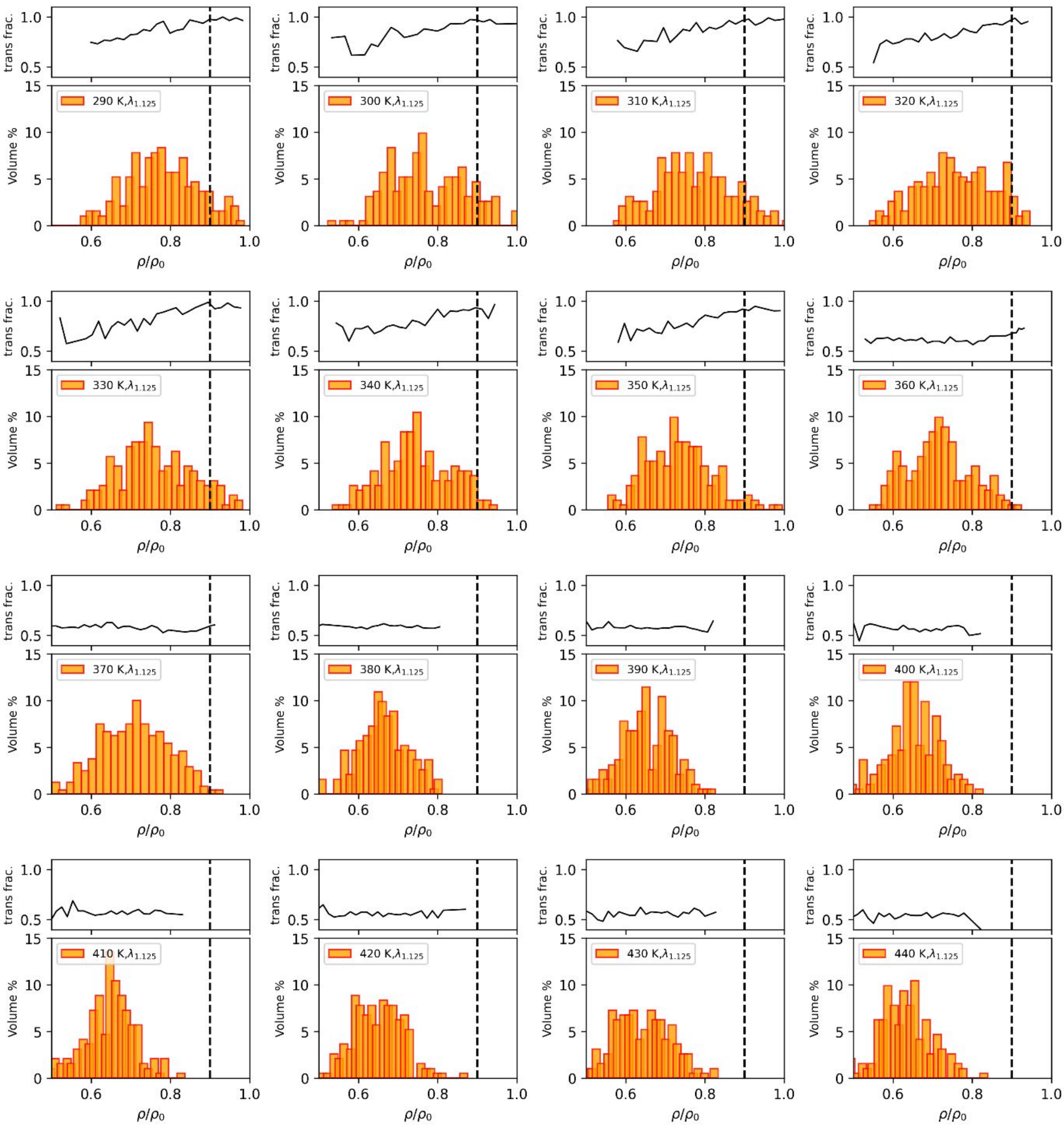

Figure SI-7. Distribution of local atomic density (ρ/ρ_0, orange histograms) and the trans fraction (upper panels) as a function of temperature (290-440 K) for an interaction strength λ = 1.125. The vertical dashed line marks the cutoff at ρ/ρ_0 = 0.9, corresponding to 90% of the ideal-crystal density for infinite linear chains in the trans-planar conformation. For each temperature, the density distribution (bottom panel) is paired with its corresponding trans-fraction distribution (top panel), enabling direct comparison between local structural order and chain conformational state across the explored temperature range.

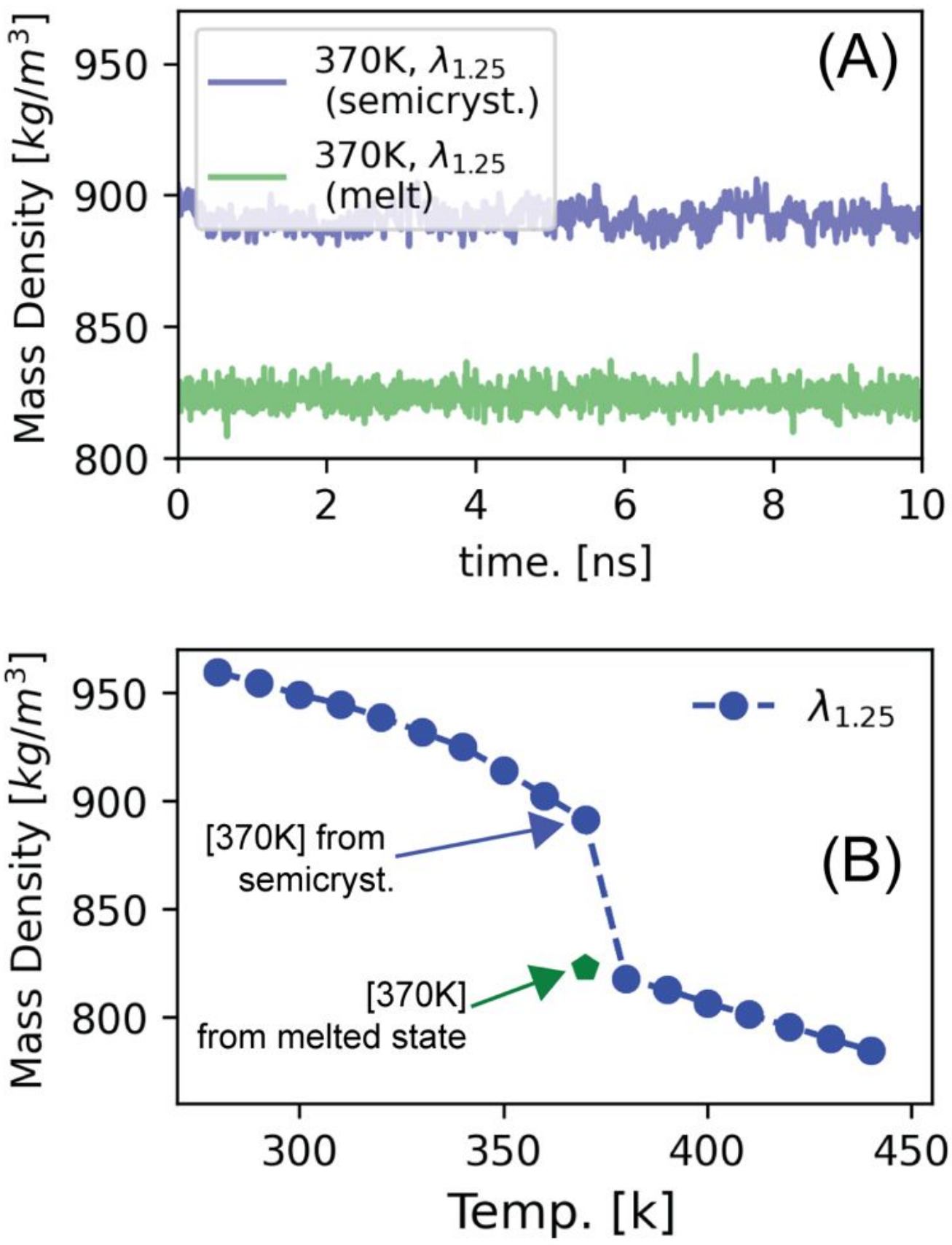

Figure SI-8. (A) Mass density of samples simulated at 370 K. The blue line shows the density of the sample in the semi-crystalline state (before melting), while the green line shows the equilibrium mass density obtained by quenching the sample after the melt memory is lost. In panel (B), the average mass density is shown on the whole temperature scan.

S8. Selective Erasure of High-Density Regions

To establish a causal, rather than merely correlational, link between the observed high-density regions and the melt memory effect, we designed a selective thermal erasure experiment. The starting configuration was the last frame of the equilibrated melt at $T = T_m = 380$ K, $\lambda = 1.25$, which retains a population of above-threshold high-density regions, as shown in Figure SI-9A (orange distribution, $t = 0$ ns).

Simulation Protocol

Chains were partitioned into two groups based on their spatial location at $t = 0$ ns:

Group 1: chains for which at least one bonded segment resides in a subcell with local density above the threshold ($\rho/\rho_0 \geq 0.9$). These chains constitute the putative carriers of melt memory.

Group 2: all remaining chains, residing exclusively in subcells below the threshold.

A dual-thermostat NPT simulation of 225 ns was then performed using the velocity-rescaling thermostat applied independently to each group. Group 1 was thermostated at $T = 420$ K, well above T_m, to selectively disrupt local chain order and erase the density contrast. Group 2 was maintained at $T = 380$ K, preserving the system's global melt state and ensuring that no bulk crystallization or global structural change occurred during the erasure procedure. Pressure was maintained at 1 atm.

Erasure of High-Density Regions

The evolution of the local density distribution was monitored at regular intervals throughout the 225 ns simulation. As shown in Figure SI-9A,B (green distribution, $t = 225$ ns), the above-threshold population is eliminated by the end of the simulation. The local density distribution of the selectively heated system at $t = 225$ ns is indistinguishable from that of a fully isotropic melt, confirming that the dual-thermostat protocol successfully erases the high-density domains without introducing artifacts in the bulk melt structure.

Cooling Experiment

To directly test whether erasure of the high-density regions abolishes the melt memory effect, the configuration obtained at $t = 225$ ns was subjected to the same slow thermal quench previously used in Figure 9D of the main text (cooling rate: 0.2 $K \cdot ns^{-1}$, from 380 K to 280 K). This cooling rate was deliberately chosen because it is the most discriminating condition

identified in Figure 9: at 0.2 $K \cdot ns^{-1}$, the intact *Domain IIa* system crystallizes clearly within the simulation timeframe, while the isotropic melt does not. As shown in Figure SI-9C, the selectively heated system fails to crystallize within the simulation timeframe. The bulk mass density remains at melt-characteristic values throughout the entire cooling run, with no abrupt increase indicative of a crystallization event. This behavior is in stark contrast to the original Domain IIa system under identical cooling conditions and is fully consistent with the behavior of an isotropic melt.

The selective erasure experiment demonstrates that removing high-density locally ordered regions, achieved without modifying the global temperature, chain architecture, force-field parameters, or overall melt structure, is sufficient to abolish the accelerated crystallization associated with melt memory. This result establishes that the presence of these domains is not merely correlated with melt memory but is causally necessary for the effect to manifest. Combined with the structural evidence presented in the main text (elevated trans fraction, partial orientational order, and chain-identity persistence across the phase transition), this experiment provides the most direct mechanistic support for interpreting high-density regions as the molecular carriers of melt memory in *Domain IIa*.

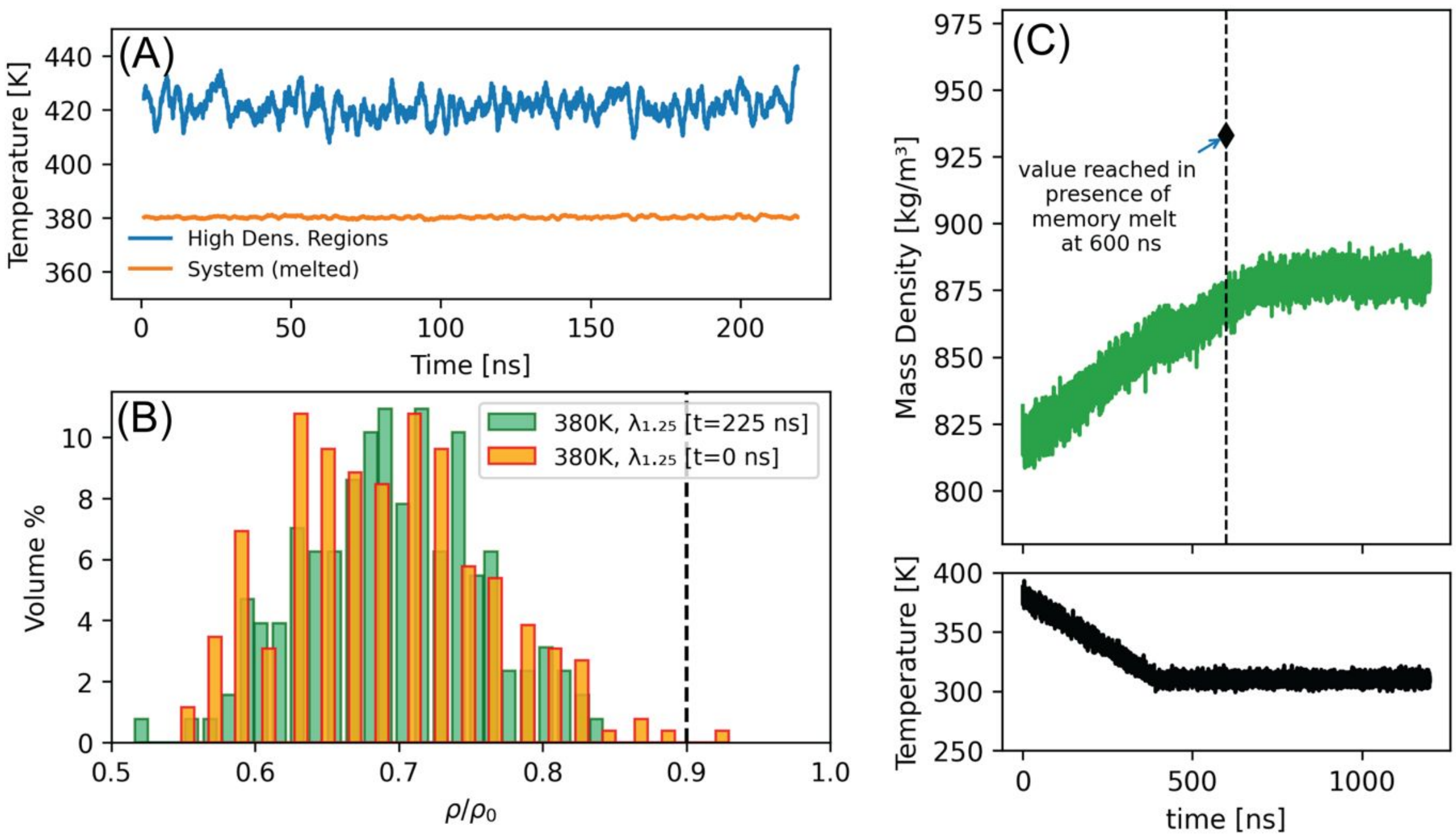

Figure SI-9. (A) Instantaneous temperature of Group 1 (blue) and Group 2 / global system (orange) throughout the 225 ns erasure simulation, confirming independent thermostating of the two chain populations. (B) Local density distributions at $T = 380$ K, $\lambda = 1.25$, at $t = 0$ ns (orange) and $t = 225$ ns (green) after dual-thermostat selective erasure of high-density regions. The dashed vertical line marks the $\rho/\rho_0 = 0.9$ threshold. (C) Time evolution of bulk mass density (upper panel) and temperature (lower panel) during slow cooling at 0.2 $K{\cdot}ns^{-1}$ applied to the configuration obtained after selective erasure. The system remains in the melt state throughout the cooling run, in contrast to the intact *Domain IIa* system reported in Figure 9D of the main text.

References

(1) Paul, W.; Yoon, D. Y.; Smith, G. D. An Optimized United Atom Model for Simulations of Polymethylene Melts. *The Journal of Chemical Physics* **1995**, *103* (4), 1702–1709. https://doi.org/10.1063/1.469740.

(2) Waheed, N.; Ko, M. J.; Rutledge, G. C. Molecular Simulation of Crystal Growth in Long Alkanes. *Polymer* **2005**, *46* (20), 8689–8702. https://doi.org/10.1016/j.polymer.2005.02.130.

(3) Yamamoto, T. Molecular Dynamics of Polymer Crystallization Revisited: Crystallization from the Melt and the Glass in Longer Polyethylene. *The Journal of Chemical Physics* **2013**, *139* (5), 054903. https://doi.org/10.1063/1.4816707.

(4) Yamamoto, T. Molecular Dynamics of Reversible and Irreversible Melting in Chain-Folded Crystals of Short Polyethylene-like Polymer. *Macromolecules* **2010**, *43* (22), 9384–9393. https://doi.org/10.1021/ma101777d.

(5) Koyama, A.; Yamamoto, T.; Fukao, K.; Miyamoto, Y. Molecular Dynamics Studies on Polymer Crystallization from a Stretched Amorphous State. *Journal of Macromolecular Science, Part B* **2003**, *42* (3–4), 821–831. https://doi.org/10.1081/MB-120021608.

(6) Ramos, J.; Vega, J. F.; Martínez-Salazar, J. Molecular Dynamics Simulations for the Description of Experimental Molecular Conformation, Melt Dynamics, and Phase Transitions in Polyethylene. *Macromolecules* **2015**, *48* (14), 5016–5027. https://doi.org/10.1021/acs.macromol.5b00823.

(7) Milano, G.; Kawakatsu, T. Hybrid Particle-Field Molecular Dynamics Simulations for Dense Polymer Systems. *The Journal of Chemical Physics* **2009**, *130* (21), 214106. https://doi.org/10.1063/1.3142103.

(8) Milano, G.; Kawakatsu, T. Pressure Calculation in Hybrid Particle-Field Simulations. *The Journal of Chemical Physics* **2010**, *133* (21), 214102. https://doi.org/10.1063/1.3506776.

(9) De Nicola, A.; Kawakatsu, T.; Milano, G. Generation of Well-Relaxed All-Atom Models of Large Molecular Weight Polymer Melts: A Hybrid Particle-Continuum Approach Based on Particle-Field Molecular Dynamics Simulations. *J. Chem. Theory Comput.* **2014**, *10* (12), 5651–5667. https://doi.org/10.1021/ct500492h.

(10) De Nicola, A.; Munaò, G.; Grizzuti, N.; Auriemma, F.; De Rosa, C.; Sevink, A.; Milano, G. Generation of Well Relaxed All Atom Models of Stereoregular Polymers: A Validation of Hybrid Particle-Field Molecular Dynamics for Polypropylene Melts of Different Tacticities. *Soft Materials* **2020**, *18* (2–3), 228–241. https://doi.org/10.1080/1539445X.2020.1716801.

(11) Zhao, Y.; De Nicola, A.; Kawakatsu, T.; Milano, G. Hybrid Particle-Field Molecular Dynamics Simulations: Parallelization and Benchmarks. *J. Comput. Chem.* **2012**, *33* (8), 868–880. https://doi.org/10.1002/jcc.22883.

(12) Abraham, M. J.; Murtola, T.; Schulz, R.; Páll, S.; Smith, J. C.; Hess, B.; Lindahl, E. GROMACS: High Performance Molecular Simulations through Multi-Level Parallelism from Laptops to Supercomputers. *SoftwareX* **2015**, *1–2*, 19–25. https://doi.org/10.1016/j.softx.2015.06.001.

(13) Caminiti, R.; Pandolfi, L.; Ballirano, P. Structure of Polyethylene from X-Ray Powder Diffraction: Influence of the Amorphous Fraction on Data Analysis. *Journal of Macromolecular Science, Part B* **2000**, *39* (4), 481–492. https://doi.org/10.1081/MB-100100400.

(14) Rai, N.; Wagner, A. J.; Ross, R. B.; Siepmann, J. I. Application of the TraPPE Force Field for Predicting the Hildebrand Solubility Parameters of Organic Solvents and Monomer Units. *J. Chem. Theory Comput.* **2008**, *4* (1), 136–144. https://doi.org/10.1021/ct700135j.
